# Partially Redundant Enhancers Cooperatively Maintain Mammalian *Pomc* Expression Above a Critical Functional Threshold

**DOI:** 10.1371/journal.pgen.1004935

**Published:** 2015-02-11

**Authors:** Daniel D. Lam, Flavio S. J. de Souza, Sofia Nasif, Miho Yamashita, Rodrigo López-Leal, Veronica Otero-Corchon, Kana Meece, Harini Sampath, Aaron J. Mercer, Sharon L. Wardlaw, Marcelo Rubinstein, Malcolm J. Low

**Affiliations:** 1 Department of Molecular and Integrative Physiology, University of Michigan Medical School, Ann Arbor, Michigan, United States of America; 2 Instituto de Investigaciones en Ingeniería Genética y Biología Molecular, Consejo Nacional de Investigaciones Científicas y Técnicas, Buenos Aires, Argentina; 3 Facultad de Ciencias Exactas y Naturales, Universidad de Buenos Aires, Buenos Aires, Argentina; 4 Centro de Estudios Científicos, Valdivia, Chile; 5 Department of Medicine, Columbia University College of Physicians and Surgeons, New York, New York, United States of America; 6 Center for Research on Occupational and Environmental Toxicology, Oregon Health & Science University, Portland, Oregon, United States of America; Stanford University School of Medicine, UNITED STATES

## Abstract

Cell-specific expression of many genes is conveyed by multiple enhancers, with each individual enhancer controlling a particular expression domain. In contrast, multiple enhancers drive similar expression patterns of some genes involved in embryonic development, suggesting regulatory redundancy. Work in Drosophila has indicated that functionally overlapping enhancers canalize development by buffering gene expression against environmental and genetic disturbances. However, little is known about regulatory redundancy in vertebrates and in genes mainly expressed during adulthood. Here we study nPE1 and nPE2, two phylogenetically conserved mammalian enhancers that drive expression of the proopiomelanocortin gene (*Pomc*) to the same set of hypothalamic neurons. The simultaneous deletion of both enhancers abolished *Pomc* expression at all ages and induced a profound metabolic dysfunction including early-onset extreme obesity. Targeted inactivation of either nPE1 or nPE2 led to very low levels of *Pomc* expression during early embryonic development indicating that both enhancers function synergistically. In adult mice, however, *Pomc* expression is controlled additively by both enhancers, with nPE1 being responsible for ∼80% and nPE2 for ∼20% of *Pomc* transcription. Consequently, nPE1 knockout mice exhibit mild obesity whereas nPE2-deficient mice maintain a normal body weight. These results suggest that nPE2-driven *Pomc* expression is compensated by nPE1 at later stages of development, essentially rescuing the earlier phenotype of nPE2 deficiency. Together, these results reveal that cooperative interactions between the enhancers confer robustness of *Pomc* expression against gene regulatory disturbances and preclude deleterious metabolic phenotypes caused by *Pomc* deficiency in adulthood. Thus, our study demonstrates that enhancer redundancy can be used by genes that control adult physiology in mammals and underlines the potential significance of regulatory sequence mutations in common diseases.

## Author Summary

The stability of animal form and function in the face of genetic and environmental variation relies on consistent gene expression. Multiple enhancers, each specifying a unique regulatory domain, control the precise spatiotemporal expression of many genes. However, in some genes apparently redundant enhancers regulate expression in overlapping cell-specific patterns. Although this arrangement has been shown to be important for developmental robustness in invertebrates, the role of apparently redundant enhancers in vertebrate species and in genes functioning in adulthood is poorly understood. Here, we show that expression of the mammalian *Pomc* gene is controlled in a tissue-specific manner by two such apparently redundant enhancers. We used targeted deletion of the individual enhancers to delineate their respective contributions to *Pomc* expression in the brain. Since *Pomc* expression from its intact *locus* exceeds the sum of the individual enhancer contributions to *Pomc* mRNA levels in embryonic mice, we infer a synergistic action between the enhancers during development. In contrast, the interaction between the enhancers is additive in adult mice. Deletion of both enhancers simultaneously almost completely abolished *Pomc* expression and the mutant mice displayed extreme obesity and metabolic dysfunction, while deletion of the individual enhancers had a modest or no phenotypic effect. Together, our results demonstrate that the two enhancers cooperatively maintain *Pomc* expression above a critical functional threshold.

## Introduction

Precise quantitative and spatiotemporal control of protein-coding gene expression is essential for normal development and cellular function. *Cis*-regulatory genomic elements, including enhancers, play pivotal roles in this control and are extensively utilized by metazoan genomes[1,2]. Although the entire repertoire of transcriptional enhancers in mammalian genomes remains to be revealed, recent genome-wide studies indicate that enhancers greatly outnumber protein-coding genes. For example, results obtained by the ENCODE project analyzing many different cell lines suggest that mammalian genomes might harbor up to 400,000 enhancer-like regions[3–5], and a recent atlas of active enhancers across human cell types and tissues has estimated that transcription is regulated by an average of 4.9 enhancers per gene[6]. Although this number may vary from gene to gene, it is clear that regulation of most of the ∼20,000 protein-coding mammalian genes is accomplished by multiple enhancers.

In many cases, each enhancer directs gene expression in particular cell types or developmental stages[7–10]. This modular organization has important evolutionary consequences, because mutations in a particular enhancer might change the expression of a gene in a particular context with no, or minor, effects in others[2]. Another emerging feature is the discovery of a number of genes regulated by more than one enhancer driving partially or completely overlapping expression patterns, suggesting regulatory redundancy. Genetic redundancy is usually related to the presence of different genes (often paralogues) performing similar functions[11–16], but regulatory redundancy is implied when different enhancers around a given gene are found to drive overlapping expression patterns in transgenic assays[17–21].

In *Drosophila melanogaster*, apparently redundant enhancers have been identified for some developmental genes and dubbed *primary* for the most proximal enhancer and *secondary* or *shadow* for the most distal enhancers[17,19,21]. Inactivation experiments of enhancer pairs present around the *shavenbaby* (*svb*) and *snail loci* suggest that each enhancer separately is dispensable for proper gene function in standard laboratory conditions, but the presence of both enhancers is essential for buffering developmental processes against environmental or genetic disturbances[20–22]. These findings indicated that redundancy between a pair of enhancers may increase phenotypic robustness and provided a molecular mechanism for the concept of “canalization” of development, first proposed by C. Waddington[11,18,19,23].

Rigorous testing of regulatory redundancy requires the inactivation of each individual enhancer and combinations of apparently redundant enhancers in their native *loci* followed by an evaluation of gene expression levels and a broad screening for differential phenotypes attributable to alteration of gene expression. In principle, such experiments might reveal either complete redundancy if a deleterious phenotype is observable only with the simultaneous inactivation of both enhancers, or partial redundancy if inactivation of each enhancer separately has a weak phenotype on its own. Since complete redundancy is thought to be evolutionarily unstable and likely to be eliminated by mutation[13], most existing cases of regulatory redundancy are expected to be of partial nature. Until now, studies on identified apparently redundant enhancers have not been truly comprehensive, as transcription levels of the endogenous genes have not been measured, genetic manipulations did not include discrete deletions of each enhancer in their native *loci* and the phenotypes have mostly been evaluated with surrogate transgenes[20,21].

In this study, we perform a comprehensive study of enhancer redundancy by taking advantage of two distal and highly conserved regulatory elements, named nPE1 and nPE2, that control hypothalamic expression of the mammalian proopiomelanocortin gene (*Pomc*). *Pomc* encodes melanocortin neuropeptides that participate in the control of food intake and body weight. The importance of *Pomc* is readily apparent in humans and mice lacking hypothalamic *Pomc* expression, which are hyperphagic and extremely obese[24–26]. The *Pomc* neuron-specific enhancers nPE1 and nPE2 drive completely overlapping spatiotemporal expression patterns of transgenic markers to the ∼3,000 POMC neurons present in the mouse ventromedial hypothalamus during embryogenesis as well as in adulthood[27,28]. In spite of their seemingly identical transcriptional specificity, nPE1 and nPE2 are not derived from a duplication but rather from the sequential exaptation (co-option) of two unrelated retroposons in the lineage leading to mammals. The more ancient enhancer, nPE2, was exapted from a CORE-SINE retroposon more than 166 million years ago (Mya) in an ancestor of all extant mammals[29], whereas nPE1 is a placental novelty originated from the co-option of a MaLR retroposon between 150 and 90 Mya[28].

Here, we provide evidence that nPE1 and nPE2, although having unrelated evolutionary origins, share a common array of homeodomain (HD)-containing transcription factor (TF) binding sites that are essential for reporter gene expression in hypothalamic POMC neurons of transgenic mice. In addition, we tackled the fundamental question of why *Pomc*, a gene primarily involved in postnatal physiology, employs two apparently redundant enhancers, instead of just one, to control hypothalamic expression. To this end, we directly investigated the contribution of each enhancer to *Pomc* expression during embryogenesis and adulthood by deleting each enhancer, or both together, from their endogenous *loci* by targeted mutagenesis. Based on the transcriptional and phenotypic consequences observed in the different mutant mice, we infer the functional and evolutionary significance of this two-component regulatory module.

## Results

### The *Pomc* Neuronal Enhancers nPE1 and nPE2 Share a Common *cis*-Regulatory Code

**Fig 1:**
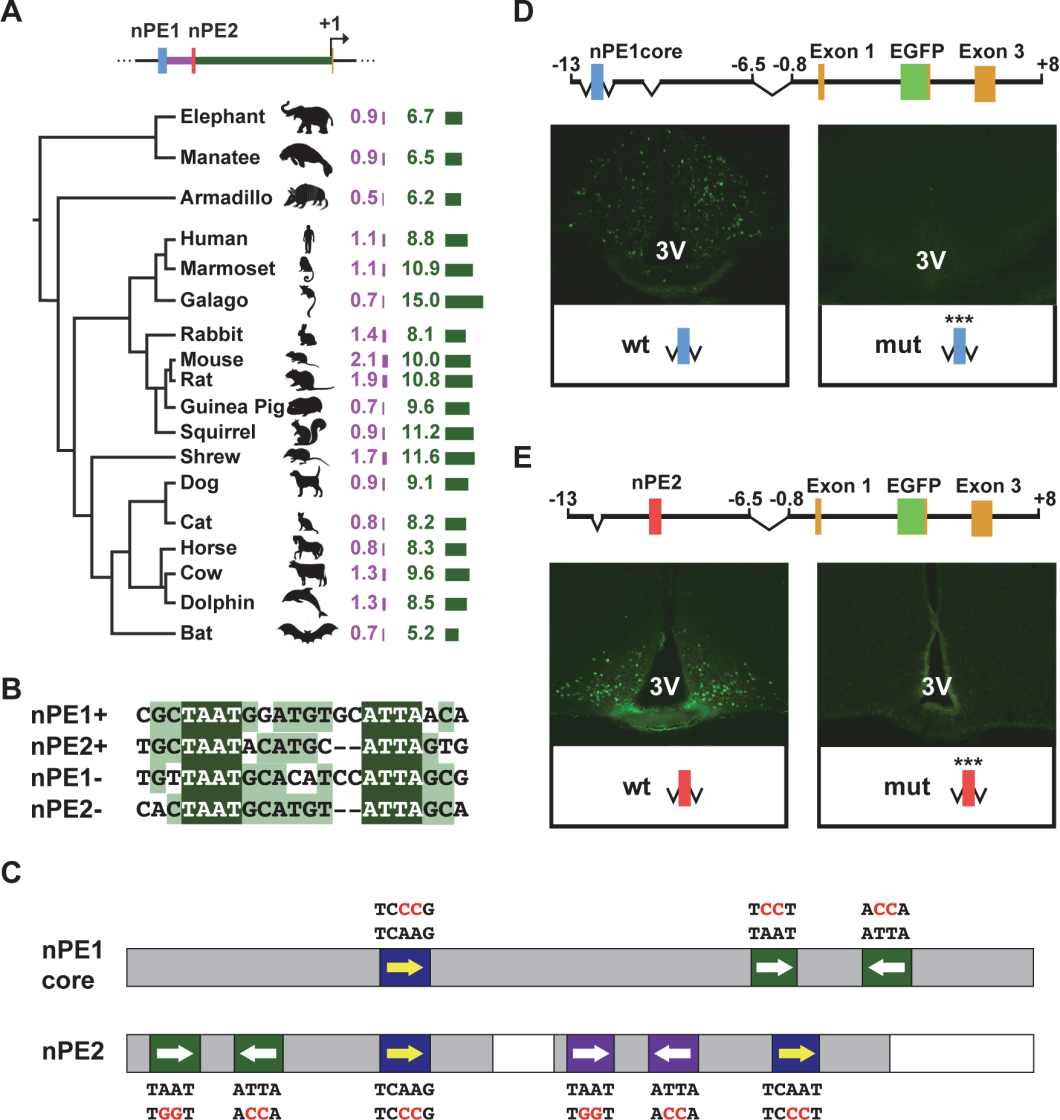
The *Pomc* neuronal enhancers nPE1 and nPE2 Share a common *cis*-regulatory code. (A) Evolutionary tree of placental mammalian lineages. The relative lengths of DNA sequences in kilobases (kb) separating nPE1 from nPE2 and nPE2 from exon 1 are illustrated by purple and green bars, respectively. (B) Alignment between an almost palindromic sequence carrying canonical homeodomain binding sites (HDBS) present in nPE1core and a remarkably similar sequence present within nPE2 region 1. (C) Scheme of nPE1core and regions 1 and 3 of nPE2 showing the relative positions of conserved HDBS. nPE1core carries a pair of inverted HDBS similar to another present in nPE2 region 1 (green boxes, full sequences depicted in Fig. 1B). Another pair of HDBS is present in region 3 of nPE2 (purple boxes). Canonical HDBS of the NKX subfamily are shown (blue boxes). Red letters indicate the mutated nucleotides. Grey boxes denote the critical enhancer regions determined previously in transgenic mice. (D) Two nearly identical transgenes were constructed to study the importance of the HDBS present in nPE1core. The control nPE1core*Pomc*-EGFP carries the wild-type (wt) enhancer sequence whereas nPE1core(mut)*Pomc*-EGFP (mut) carries six nucleotide substitutions covering all HDBS (red letters in Fig. 1C). Coronal brain sections showing EGFP expression in hypothalamic arcuate nucleus of nPE1core*Pomc*-EGFP (Left) but not in nPE1core(mut)*Pomc*-EGFP transgenic founder newborn mice (Right). (E) Two nearly identical transgenes were constructed to study the importance of the HDBS present in nPE2. The control transgene nPE2*Pomc*-EGFP carries the wild-type (wt) enhancer whereas nPE2(mut)*Pomc*-EGFP (mut) carries twelve nucleotide substitutions covering all HDBS (red letters in Fig. 1C). EGFP is expressed in the arcuate nucleus of founder transgenic mice carrying nPE2*Pomc*-EGFP (Left) but not nPE2(mut)*Pomc*-EGFP (Right). 3V, third ventricle.

Hypothalamic enhancers nPE1 and nPE2 are phylogenetically conserved in placental mammals and were initially discovered by local alignments of mouse and human *Pomc* 5′-flanking sequences[27–29]. The distance between the two enhancers and between nPE2 and *Pomc* exon 1 are also strongly conserved across placental genomes (Fig. 1A). In mice, nPE1 and nPE2 are 2.1 kb apart, constituting a two-enhancer distal regulatory module located 10.0 kb upstream of the transcriptional start site[27]. The inter-enhancer distance ranges between 0.5 and 1.9 kb in other species, whereas the distance between nPE2 and exon 1 ranges between 5.2 and 15.0 kb (Fig. 1A).

The fact that both nPE enhancers drive expression to the same population of hypothalamic neurons[27,28] suggests that these two functional analogues might share DNA elements for the binding of similar transcription factors (TFs). We searched the functionally critical regions present in both enhancers, nPE1core[28] and regions 1 and 3 of nPE2[29], for common DNA motifs and found a 21-bp imperfect palindromic sequence present in nPE1core that is highly similar to a sequence present in region 1 of nPE2 (Fig. 1B). Each sequence contains two inverted TAAT (ATTA) motifs typically recognized by HD-TFs (Fig. 1C, green boxes). Another sequence present in the critical region 3 of nPE2 also shows a similar configuration with two inverted HD binding sites (Fig. 1C, purple boxes). These similar arrays of inverted TAAT motif pairs are completed with a canonical TCAAG/T motif potentially recognized by HD-TFs of the NKX subfamily (Fig. 1C, blue boxes). Interestingly, all these HD-binding sites are conserved in humans, mice and most other mammals (S1–S2 Figs.).

The onset of neuronal *Pomc* expression at e10.5 coincides with the early patterning and initial cell-type specification of the mouse ventral hypothalamus. Because these developmental programs involve several HD-TFs we decided to investigate the importance of the HD binding DNA elements in the function of nPE1core and nPE2. To this end we constructed transgenes carrying transition mutations of these motifs in either nPE1*core* or nPE2 and screened their activity in transgenic founder mice. All transgenes carried the whole mouse *Pomc* transcriptional unit (including the proximal promoter, exons and introns) and the EGFP reporter gene inserted within *Pomc* exon 2. Control transgenes carrying either intact nPE1*core* or nPE2 drove expression to the arcuate nucleus of the hypothalamus (Fig. 1D and 1E) as previously shown [28]. In contrast, all transgenic founder mice carrying either a mutated nPE1*core* (ten independent lines) or a mutated nPE2 (three independent lines) failed to drive EGFP expression to this brain region (Fig. 1D and 1E). These results suggest that a common array of *cis* elements, possibly binding the same TFs, underlies the functional analogy of nPE enhancers.

### Targeted Inactivation of *Pomc* Enhancers

**Fig 2:**
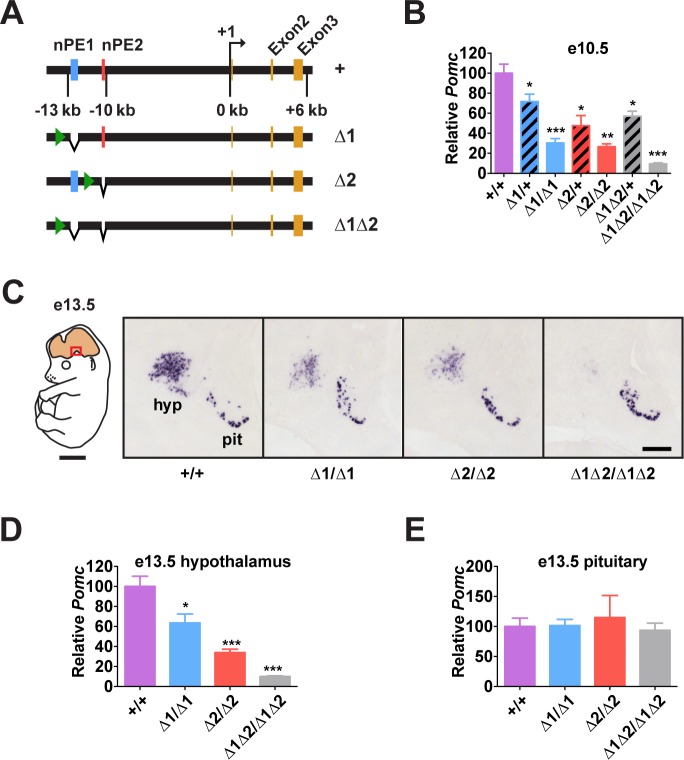
*Pomc* enhancers nPE1 and nPE2 function cooperatively. (A) Schematic of nPE mutant *Pomc* alleles. Green arrowheads show the locations of remnant loxP sites following Cre-mediated excision of the *neo* gene-targeting selection cassettes (see S3 Fig.). (B) *Pomc* expression measured by qRT-PCR in heads of e10.5 embryos. n = 7–8. (C) Representative *Pomc in situ* hybridization in sagittal sections of e13.5 embryos. Red box in schematic diagram indicates magnified area. hyp, hypothalamus; pit, pituitary. Scale bar in left panel, 2 mm; right panel, 500 μm. (D-E) Average integrated density of *in situ* hybridization signal in hypothalamus (D) and pituitary (E). n = 4. Quantitative data are presented as mean + 1 S.E.M. Genotype means were compared by two-tailed t-tests. * *P* < 0.05, ** *P* < 0.01, *** *P* < 0.001 compared to +/+.

To understand the relative importance of each enhancer for *Pomc* expression and adult physiology, we generated mouse lines carrying small deletions that eliminate nPE1 (Δ1), nPE2 (Δ2) or both enhancers (Δ1Δ2) by targeted mutagenesis (Figs. 2A and S3). The precise deleted sequences encompass the conserved 579 bp for nPE1 and 172 bp for nPE2, and are identical to the deletions we used previously to study the enhancers in transgenic experiments[27]. Importantly, the genomic DNA sequences between the two enhancers remained intact in the mutant *Pomc* alleles. The neomycin resistance cassette, required for clonal selection of targeted embryonic stem cells, was ultimately removed from the targeted loci using Cre/loxP recombination, so that the proximal promoter region, any other potential regulatory elements and the entire coding region of *Pomc* were left intact. In particular, since pituitary expression of *Pomc* depends on the proximal promoter and an enhancer located 7 kb upstream of the gene[30], we expected *Pomc* expression to be unaffected in the pituitary gland of the nPE mutant mice.

### Enhancers nPE1 and nPE2 Act Synergistically During Early Development

*Pomc* expression starts in the prospective anterior hypothalamus during embryogenesis, at e10.5[29,31,32]. At this stage, we observed that embryos lacking both enhancers had very low levels of *Pomc* mRNA (∼10% of wild-type levels, Fig. 2B), showing that nPE1 and nPE2 are responsible for most hypothalamic expression of *Pomc* in embryos. The same result was observed at e13.5 (Fig. 2C and 2D). On the other hand, embryos with homozygous deletion of either enhancer alone had a substantial reduction in hypothalamic *Pomc* expression at e10.5 (∼25% of wild-type levels, Fig. 2B), as well as at e13.5 (Fig. 2C and 2D). A significant reduction in *Pomc* mRNA was apparent even in embryos heterozygous for each enhancer deletion (Fig. 2B). We believe that reduced levels of *Pomc* transcription in every POMC neuron, rather than a loss of POMC neurons themselves, explains all these results. Previously we showed that compound mutant mice expressing a cell-autonomous POMC-EGFP reporter transgene on a background of endogenous *Pomc* deficiency in the hypothalamus had the same number of POMC neurons as wildtype mice or single POMC-EGFP transgenic mice[26]. Interestingly, *Pomc* mRNA levels transcribed from the wild-type *locus* during development are much higher than the sum produced by each single enhancer homozygous mutant, indicating that nPE1 and nPE2 act synergistically to overcome transcriptional inertia at the onset of hypothalamic *Pomc* expression. As expected, pituitary expression of *Pomc* was not affected by the deletion of any enhancer (Fig. 2C and 2E).

### Additive Interaction between nPE1 and nPE2 in Adulthood

**Fig 3:**
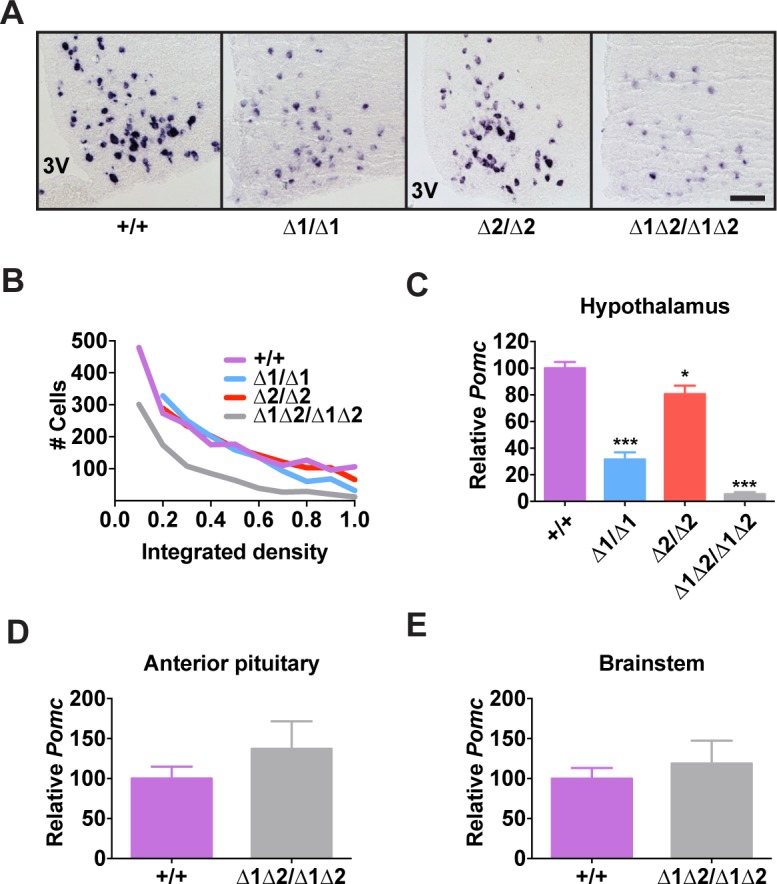
*Pomc* expression in adult nPE mutant mice. (A) Representative *Pomc in situ* hybridization with a digoxigenin-labeled riboprobe in coronal sections through the hypothalamic arcuate nucleus of adult male mice (age 8 wk). 3V, third ventricle. Scale bar, 100 μm. (B) Histogram of integrated density of the cellular colorimetric *in situ* hybridization signal. Images from a minimum of 4 sections per biological replicate were thresholded for minimum object size and intensity, and automated cell counts, together with their individual integrated optical densities, were performed by the NIS Elements software (Nikon). (C-E) *Pomc* expression measured by qRT-PCR in hypothalamus (C), anterior pituitary (D), and brainstem (E) of adult male mice (age 8 wk). n = 8 per genotype. All quantitative data are presented as mean + 1 S.E.M. Genotype means were compared by two-tailed t-tests. * *P* < 0.05, *** *P* < 0.001 compared to +/+.

**Fig 4:**
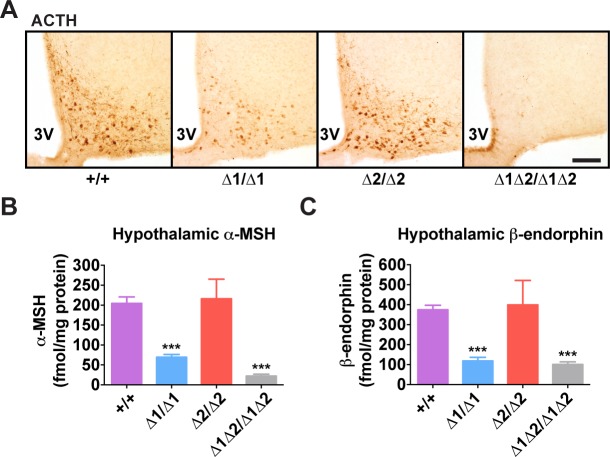
Hypothalamic POMC-derived peptides in nPE mutant mice. (A) Representative ACTH immunohistochemistry in coronal hypothalamic sections. 3V, third ventricle. Scale bar, 200 μm. (B) Hypothalamic α-MSH and (C) β-endorphin content measured by radioimmunoassay. All data are from adult male mice (age 8 wk). n = 6–18. All quantitative data are presented as mean + 1 S.E.M. Genotype means were compared by two-tailed t-tests. *** *P* < 0.001 compared to +/+.

In adult mice, the simultaneous lack of both enhancers reduced *Pomc* mRNA levels to ∼10% of wild-type levels, similar to what was observed during embryogenesis (Fig. 3A-C). The individual enhancer deletions demonstrated that each enhancer was independently able to support transcription in a full complement of hypothalamic POMC neurons, but with reduced transcriptional strength (Fig. 3A-C). Mice lacking nPE1 expressed approximately 30% of wild-type levels of *Pomc* mRNA in the hypothalamus (Fig. 3A-C), similar to that observed in embryos. However, mice lacking nPE2 expressed only ∼20% less *Pomc* mRNA than wild-type controls (Fig. 3A-C), indicating that relative expression levels increased from 25% of wild-type levels to 80% in nPE2-null mice during the period between embryogenesis and adulthood (compare Fig. 2B and 2D with Fig. 3C). Consistent with their hypothalamic cell-specific enhancer activity, deletion of the enhancers did not affect *Pomc* expression in corticotrophs of the anterior pituitary (Fig. 3D) or brainstem neurons (Fig. 3E). Levels of POMC-derived peptides in the arcuate nucleus as well as brain regions receiving afferent peptidergic inputs from *Pomc*-expressing neurons were commensurate with *Pomc* mRNA levels (Figs. 4 and S4).

### Adult Mice Lacking nPE1 Display Overweight and an Abnormal Metabolic Phenotype

**Fig 5:**
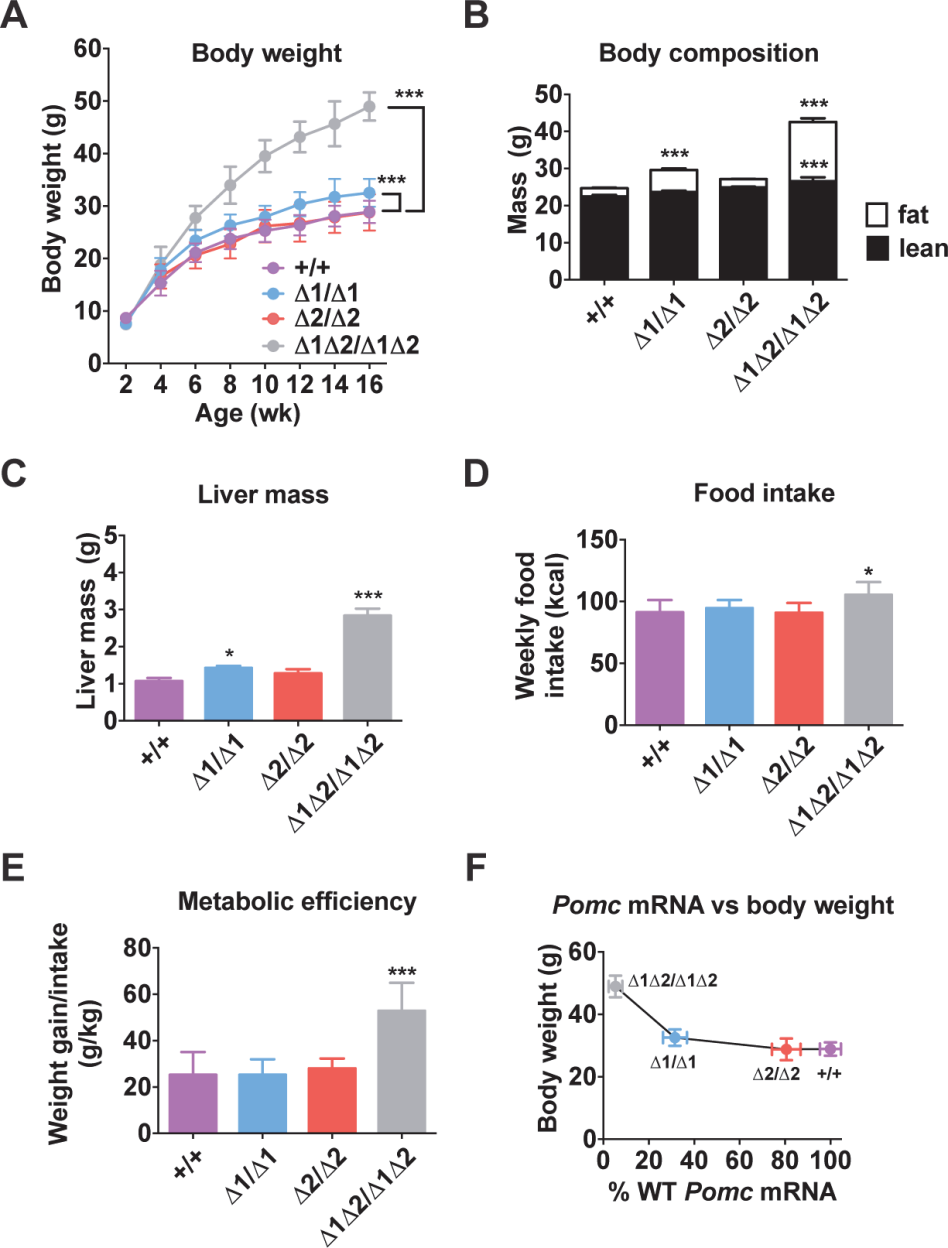
Metabolic phenotype of male nPE mutant mice. (A) Body weight. n = 5–11. (B) Body composition measured by NMR of mice age 16 wk. n = 15. (C) Liver mass measured in mice aged 16 wk. n = 6. (D) Food intake and (E) metabolic efficiency measured for 1 wk (age 6–7 wk). n = 5–9. (F) Body weight data from (A) plotted against *Pomc* mRNA data from Fig. 3C. All quantitative data are presented as mean ± 1 S.E.M. Genotype means were compared by two-tailed t-tests. * *P* < 0.05, *** *P* < 0.001 compared to +/+.

An analysis of body weight and food intake of enhancer-deficient adult mice revealed a threshold effect of *Pomc* transcription on phenotype. Animals lacking both enhancers were severely obese, hyperphagic and hypometabolic, all features consistent with their low levels of hypothalamic *Pomc* expression (Fig. 5A-E). Mice lacking nPE1, which still expressed 30% of wild-type *Pomc* levels, had unaltered food intake but displayed moderate weight gain and obesity based on increased total fat mass and liver mass, consistent with steatosis (Fig. 5A-C). In contrast, mice lacking nPE2 were able to maintain normal food intake, body weight and composition, consistent with their expression of *Pomc* mRNA at levels close to the wild-type controls (Fig. 5A-C). Plotting *Pomc* expression against body weight reveals the existence of a threshold at ∼30% of wild-type levels, below which the shallow linear relationship between hypothalamic *Pomc* expression and body weight is abruptly altered (Fig. 5F), as previously suggested in cytotoxic POMC neuron lesion studies[33].

### Adult Mice Lacking nPE2 Display a Normal Metabolic Phenotype

**Fig 6:**
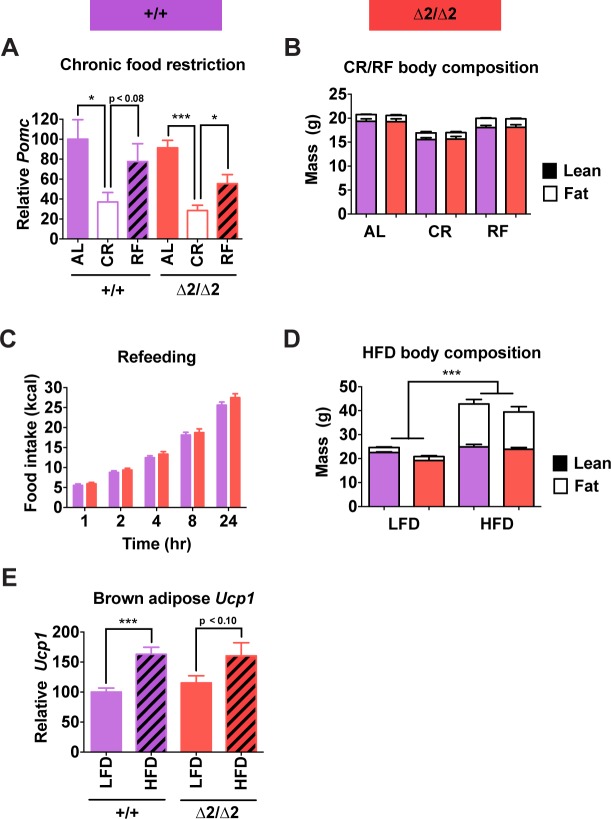
Physiological responses of male nPE2 mutant mice to altered nutrition. (A) *Pomc* expression measured by qRT-PCR in hypothalamus and (B) body composition measured by NMR of mice fed *ad libitum* (AL), fed a calorie restricted diet causing 20% weight loss in 1 wk (CR), or subjected to CR regimen followed by 24 hr *ad libitum* food access (RF). n = 6 of each genotype/treatment combination. (C) Cumulative food intake at each indicated time point during the 24 hr refeeding period. (D) Body composition measured by NMR in mice fed low fat (LFD; 10% kcal fat) or high fat (HFD; 60% kcal fat) diets for 16 wk. n = 2 for LFD, 6–7 for HFD. (E) *Ucp1* expression in brown adipose tissue measured by qRT-PCR of mice fed LFD or HFD for 48 hr. n = 6 per treatment/genotype combination. Data are presented as mean + 1 S.E.M. Group means were compared by two-tailed t-tests. * *P* < 0.05, *** *P* < 0.001.

The preceding experiments were all performed using mice fed a standard low fat chow *ad libitum*. Therefore, we questioned whether a latent altered phenotype of adult nPE2 knockout mice would be revealed by either chronic calorie restriction or surfeit. However, nPE2 knockout mice showed a normally decreased *Pomc* transcriptional response to two-week food restriction, followed by a brisk rebound after 24 hr refeeding (Fig. 6A). The mice also exhibited similar changes in body composition and compensatory refeeding responses compared with wild-type controls (Fig. 6B and C). Chronic high fat diet for 16 weeks resulted in similar increases in body weight and total body fat for nPE2 knockout and wild-type mice (Fig. 6D), while acute high fat diet exposure for two days induced similar increases in uncoupling protein 1 (*Ucp1*) mRNA levels in brown adipose tissue of both genotypes (Fig. 6E).

Furthermore, there were no observable phenotypic abnormalities in adult nPE2 knockout mice of either sex compared to wild-type controls in numerous other physiological parameters. These additional tests of reproductive capacity, glucose homeostasis, stress responses, acute fasting, food-oriented behavior, energy expenditure, cardiovascular function, and cold adaptation (S5–S6 Figs.) were designed to probe for more subtle or environment-specific phenotypic alterations in response to deletion of the evolutionarily older *Pomc* enhancer. Altogether, these results show that while nPE1 is a fundamental neuronal *Pomc* enhancer at all mouse ages, the relative contribution of the evolutionarily more ancient enhancer nPE2 to *Pomc* hypothalamic expression declines after mouse development and is only critical in adult mice lacking nPE1. Therefore, the sole presence of nPE1 in adults suffices to maintain up to 85% of *Pomc* mRNA levels and prevent metabolic dysfunction.

## Discussion

In this study, we investigated the molecular and functional relationships of the apparently redundant neuron-specific *Pomc* enhancers nPE1 and nPE2 by evaluating the effects of precise targeted deletions of each enhancer, or both at the same time, on *Pomc* expression during embryonic and postnatal life and on physiological phenotype in adulthood. Our results indicate that (i) nPE1 and nPE2 share a common set of DNA motifs that are functionally critical for their enhancer activity; (ii) *Pomc* expression depends on both enhancers, since mRNA levels drop precipitously in mice lacking both nPE1 and nPE2; (iii) at early stages of development, the two enhancers act synergistically to maintain normal *Pomc* expression levels, since the level of *Pomc* mRNA in wild-type embryos greatly exceeds that achieved by the sum of the individual enhancer mutants; (iv) in adulthood, however, the enhancers act additively in driving *Pomc* transcription at wild-type levels; (v) nPE1 inactivation revealed its predominant contribution to the overall level of adult *Pomc* expression since its absence causes several metabolic phenotypes including obesity; and (vi) deletion of nPE2 does not cause any overt physiological alteration in adult mice but precipitates hyperphagia and extreme obesity if nPE1 is simultaneously absent. However, It is important to note that despite the body of evidence implicating nPE1 and nPE2 as major contributors to *Pomc* transcription in hypothalamic neurons, our studies do not conclusively rule out the presence of additional unidentified enhancers because *Pomc* mRNA is not completely absent in mice lacking both nPE1 and nPE2.

Redundancy is a well-known phenomenon in genetics. Genomes of yeast, plants and animals have many gene paralogues that are remnants of past gene duplications or even whole-genome duplications[34]. Many such gene duplicates have overlapping functions, as evidenced by gene-inactivation experiments showing that lack of one gene can be compensated by its paralogue[16]. Redundancy of regulatory regions is a much less studied phenomenon, because *cis*-acting elements have rarely been inactivated in their native locus to study phenotypic changes. For instance, the loss-of-function studies of apparently redundant enhancers of the *Drosophila* gene *snail* were performed using large BAC transgenes, and the functional analyses of the enhancers were performed by rescuing gastrulation defects in *snail* mutants with BAC constructs[21]. In another study on the fly *svb* gene, two apparently redundant enhancers out of five were eliminated by a broad 32 kb deletion in the native *locus*[20]. However, the reciprocal deletion (the other three remaining *svb* enhancers) or the individual inactivation of each enhancer was not investigated. In contrast to these recent *Drosophila* experiments, in the present study we precisely disrupted each mouse *Pomc* enhancer in its native genomic *locus*. Importantly, we measured the amount of endogenous *Pomc* mRNA produced as well as the metabolic phenotypes associated with each targeted mouse line, yielding a quantitative assessment of the extent of functional redundancy between nPE1 and nPE2.

*Pomc* is in many ways an ideal gene in which to study enhancer redundancy. Its expression pattern is simple since it is mainly restricted to the pituitary and the hypothalamus. Furthermore, the regulatory regions controlling transcription in each of these tissues are well known. Pituitary expression is driven by the proximal promoter and an enhancer located around −7 kb upstream of the transcription start site, also suggesting redundancy[30], while neuronal expression is driven by a distal module containing nPE1 and nPE2. The hypothalamic enhancers are conserved in all placental mammals both in terms of nucleotide sequence as well as in organization within the locus, strongly suggesting that both enhancers play important roles in *Pomc* expression and mammalian physiology. In contrast, the identity of regulatory sequences responsible for the relatively low levels of *Pomc* expression in brainstem neurons and skin have yet to be identified.

In molecular terms, what makes two highly distinct enhancers drive expression to the same cell type? The group of M. Levine identified what they called “primary” and “shadow” enhancers in the vicinities of a few *Drosophila* genes based on their common binding to the transcription factors Dorsal, Twist and Snail[17]. Previously we found a functional nuclear receptor binding site in nPE2, but this site is not present in nPE1[35]. Here, in contrast, we identified DNA sequence motifs shared by both nPEs that are absolutely necessary for transgene expression in the mouse hypothalamus. Therefore, it is likely that nPE1 and nPE2 interact with a similar set of yet unidentified transcription factors, in concordance with the few redundant enhancers previously described in *Drosophila*. This hypothesis would explain the overlapping spatiotemporal activities of nPE1 and nPE2. The cognate TFs are likely to already be present at e10.5 in the developing mouse ventral forebrain, as *Pomc* is one of the first neuropeptide genes to be expressed in the prospective hypothalamus. Our identification of the common *cis*-code of nPE enhancers in this report will facilitate the search for transcription factors controlling hypothalamic *Pomc* expression.

The phylogenetic conservation of the hypothalamic *Pomc* enhancers in all placental mammals, both in terms of nucleotide sequence as well as in organization within the *locus*, strongly suggest that they play important roles in *Pomc* transcription and mammalian physiology. Our results show that, when both enhancers are deleted, *Pomc* transcription during embryogenesis and adulthood proceeds at very low levels (10% of wild-type), leading to severe metabolic dysfunction in the mutant mice. The phenotype includes hyperphagia, decreased energy expenditure and early-onset obesity, in line with previous reports of neuronal-specific *Pomc* deficiency in mice[26,36]. This shows that nPE1 and nPE2 are indeed critical for hypothalamic *Pomc* expression and illustrates the usefulness of phylogenetic conservation to identify regulatory regions of functional importance[27–29].

To examine the level of functional redundancy of nPE1 and nPE2, we have analyzed their interplay at two different, although related, levels: transcriptional efficiency and physiological phenotypes associated with *Pomc* function in adult animals. From the point of view of enhancer activity, our results indicate that the extent of functional overlap between the enhancers changes as development progresses. At the onset of *Pomc* expression (e10.5), the enhancers cooperate in a synergistic fashion, since the lack of either enhancer reduces *Pomc* mRNA to 25–30% of wild-type levels. Similar results were obtained at e13.5, when the number of POMC neurons reaches its peak[37]. In adulthood, however, the effect of each enhancer knockout changes: while lack of nPE1 still reduces *Pomc* mRNA to 30% of wild-type level, the lack of nPE2 reduces *Pomc* mRNA only to 80% of wild-type levels. Thus, in adulthood the separate activities of each enhancer are simply additive with ∼80% of the activity being due to nPE1 and ∼20% to nPE2. This observation is in agreement with a recent genome-wide survey performed by the FANTOM5 project, which found a positive correlation between the number of redundant enhancers and the expression levels of putative target genes[6]. The process of recruiting multiple enhancers to increase expression levels could be regarded as a mechanism of “superfunctionalization” or “reinforcement” of regulatory elements, akin to processes like the multimerization of genes to increase expression levels, as observed for clock genes in some bacteria[38] and ribosomal genes in eukaryotes.

The precise molecular mechanism(s) responsible for the differential contributions of nPE1 and nPE2 to *Pomc* transcriptional activation in the embryonic and adult hypothalamus have yet to be determined. It is plausible that distinct combinations of TFs and/or co-activators are recruited to the enhancer locus as POMC neurons progress from their early developmental commitment and differentiation to final maturation. Alternatively, pioneer TFs that require both enhancers may be responsible for chromatin remodeling at the onset of *Pomc* gene activation, followed by permanent epigenetic changes that bias enhancer usage to nPE1. These possibilities are not mutually exclusive and further experiments are needed to define the actual mechanism.

Concerning the effects on adult physiology, the results differ for each enhancer knockout. nPE1 inactivation caused moderate overweight and increased total body fat. The inactivation of nPE2, on the other hand, caused no discernible phenotype in a comprehensive panel of experiments analyzing metabolic parameters. Strictly speaking nPE2 is not fully redundant, because *Pomc* mRNA levels are lower in adult mutants than in wild-type individuals. However, from a physiological point of view its functions appear to be fully compensated by nPE1, at least in a modern mouse barrier facility with an *ad libitum* feeding regimen. This report together with our own previous studies demonstrates that hyperphagia and overweight are evident once *Pomc* mRNA levels drop below ∼30–40% of normal values (see Fig. 5F). Thus, although a 20% reduction in *Pomc* mRNA level observed in nPE2 knockout mice does not seem to alter body weight regulation, this decrease brings values closer to the threshold below which satiety control is impaired. The evolutionary imperative to maintain *Pomc* expression above this threshold is clear. Hyperphagia and obesity are highly maladaptive in the wild, since predator exposure is increased in hyperphagic animals due to increased foraging, while their greater mass increases visibility, impairs escape and limits reproductive success[39]. Although the mechanisms are not completely understood, obesity is also associated with decreased fertility in both men and women[40,41].

Interestingly, lack of nPE2 function is actually rescued as development progresses, since *Pomc* mRNA in nPE2 homozygous knockout embryos corresponds to only 25% of wild-type levels. If mutants were to reach adulthood expressing this decreased amount of *Pomc* mRNA, the mice would exhibit several deleterious metabolic phenotypes, as the nPE1 knockouts indicate. Instead, *Pomc* mRNA levels of nPE2 adult mutants are restored to 80% of wild-type. Thus, regulatory redundancy leads to an adjustment in the levels of *Pomc* transcription during development into adulthood in the case of functional impairment of nPE2, an observation which is reminiscent of the idea of “canalization” as proposed by C. Waddington[23]. Canalization leads to robustness in development against environmental and genetic disturbances, something that has been proposed to be an evolutionary explanation behind the existence of apparently redundant enhancers in *Drosophila*[18–21]. In the case of *Pomc*, our results indicate that the presence of the more recently evolved enhancer nPE1 can secure a normal phenotype in mice deficient in the more ancient enhancer nPE2. This functional rescue, however, is not reciprocal.

What are the possible perturbations that may alter nPE enhancer activity in the wild that would need to be canalized? On the one hand, there might be alterations in the amounts of critical TFs for *Pomc* expression either by genetic background effects or environmental conditions. On the other hand, there might be mutations (single-base changes or small indels) in the enhancers that affect TF binding. Our observation that the activities of nPE1 and nPE2 likely depend on a common set of TFs suggest that perturbations in regulatory inputs will affect the activity of both enhancers at the same time (instead of only one as in knockouts), and we hypothesize that in these situations the presence of two partially redundant enhancers should serve to maintain *Pomc* transcription above a critical threshold to avoid deleterious metabolic phenotypes.

Finally, our results highlight the fact that the only way of fully evaluating the contributions of individual enhancers to transcription is to perform comprehensive inactivation experiments in their native genomic context and then to study the physiology and fitness of the individual mutants. In recent years, genome-wide surveys have identified thousands of genomic regions with chromatin signatures indicative of potential enhancer activity[6,42], but the presence of chromatin marks or transgene assays are insufficient to conclusively assign regulatory functions to a particular genomic region, particularly for regions that are not phylogenetically conserved[2]. Genome-wide association studies (GWAS) have found that genome variants linked to human diseases are often located in the non-coding portion of the genome, indicating that many polymorphisms in enhancers may contribute to disease[43,44]. Regions near the *Pomc* locus have been implicated in predisposition to obesity and related traits[45–47], and our work shows that any variant in the nPE enhancers or distant regions that establish contacts with the enhancer module might influence *Pomc* expression. In any event, our work indicates that enhancer redundancy increases the challenges of studying the physiological significance of regulatory variation, as has been suggested for phenotypic robustness in general[48]. Hopefully, methods that permit the study of the regulatory landscape of whole loci[49,50] and newly-developed technologies that expedite genome editing[51,52] will accelerate the understanding of the prevalence and extent of regulatory redundancy and robustness in mammalian genomes.

## Materials and Methods

### Animal Care

All experiments were approved by the University of Michigan University Committee on the Care and Use of Animals (UCUCA) and followed the Public Health Service guidelines for the humane care and use of experimental animals. Mice were housed in ventilated cages under controlled temperature and photoperiod (12-hr light/12-hr dark cycle, lights on from 06:00 to 18:00 with tap water and laboratory chow containing 28.0% kcal protein, 12.1% kcal fat, and 59.8% kcal carbohydrate available *ad libitum*, except where noted otherwise.

### Bioinformatics

*Pomc loci* of mammalian genomes were identified by BLAST searches in the Ensembl website (http://www.ensembl.org) and nPE1 and nPE2 sequences were aligned with CLUSTAL W[53].

### Transgene Construction and Transgenic Mice Analysis

Transgenes nPE1*Pomc*-EGFP, nPE1core*Pomc*(mut)-EGFP, nPE2*Pomc*-EGFP and nPE2*Pomc*(mut)-EGFP were constructed using standard molecular biology techniques. The transgenes are similar to transgene 2 in ref. [27]. They encompass from −13 to +8 kb around the mouse *Pomc* locus with the deletion of a region flanked by two SmaI sites located at −6.5 and −0.8 kb. The deletions of nPE1 in nPE2*Pomc*-EGFP and nPE2 in nPE1*Pomc*-EGFP are exactly the same as those described previously for transgenes 7–12 in Fig. 5 of ref. [27]. The transgenes include the three exons of mouse *Pomc* and the coding region of EGFP inserted into a StuI site present in exon 2, before the ATG translation start codon, as previously described[27]. Parental constructs nPE1*Pomc*-EGFP and nPE2*Pomc*-EGFP were assembled as previously described[27,28] and mutations to generate the mutant version (mut) of each enhancer were introduced using standard megaprimer PCR procedures. For transgene nPE1core*Pomc*(mut)-EGFP, naturally occurring *Bst Z17I* and *Hind III* sites were used to replace the nPE1core WT sequence with the mutated 6-bp sequence shown in Fig. 1C. A similar strategy was used to construct nPE2*Pomc*(mut)-EGFP where the nPE2 wild-type sequence from the parental construct[28] was replaced using naturally occurring *Sph I* and *Xba I* sites. Transgenic mice were generated by pronuclear microinjection of B6CBF2 zygotes as described previously[27,28] at the University of Michigan Transgenic Animal Model Core Facility (Ann Arbor, Michigan, USA) and the Transgenic Mouse Unit of the Centro de Estudios Científicos (Valdivia, Chile). Newborn founder transgenic mouse brains were fixed in 4% paraformaldehyde (PFA) overnight and then cryoprotected in 30% sucrose in PBS for an additional 48 h. Coronal 30 μm brain sections were cut with a cryostat and hypothalamic EGFP expression was scored in mice showing positive transgenic signal in melanotropes of the pituitary intermediate lobe. Sections were visualized directly or immunostained with the primary polyclonal rabbit anti-EGFP (Abcam, ab290) followed by a secondary anti-rabbit Alexa Fluor 488 (A11008, Life Technologies).

### Generation and Breeding of nPE Mutant Mice

Two targeting vectors were constructed with genomic sequences between −13 and −6.5 kb of mouse *Pomc* that were isolated previously[54] and subcloned fragments harboring both a 579 bp deletion of nPE1 and a 172 bp deletion of nPE2, or the individual nPE2 deletion[27]. A neomycin-resistance cassette (PGK-neo-bGHpolyA) flanked by loxP sites was inserted into an *Apa*I site located upstream of the deleted nPE1 region (common fneoΔ1 and fneoΔ1Δ2 construct) or into an *Sph*I site located upstream of the deleted nPE2 region (fneoΔ2 construct). The 5’ and 3’ recombination arms for the fneoΔ1Δ2 construct encompassed 1.8 kb and 9.5 kb, respectively, and for the fneoΔ2 construct the 5’ and 3’ recombination arms encompassed 3.0 kb and 3.3 kb, respectively. Each targeting vector also included a *Herpes simplex* I thymidine kinase expression cassette (HSV-TK) adjacent to one of the recombination arms to enrich for homologous recombination events over random chromosomal integrations. The targeting vectors were linearized with *Kpn*I and electroporated into 129/SvJae J1 ES cells[55] or 129S6/SvEvTac Taffy ES cells (fneoΔ1Δ2 construct only; University of Cincinnati Gene Targeting and Transgenic Mouse Models Core), which were then propagated under positive-negative selection with G418 and gancyclovir. Individual clones were screened for correct homologous recombination across both arms of the targeting vectors by Southern blot analysis of genomic DNA digested with *Eco*RV. Membranes were hybridized separately to one of five unique [^32^P]-radiolabeled probes cloned by PCR from mouse genomic DNA (S3 Fig.). The fneoΔ1Δ2 allele and the fneoΔ1 alleles resulted from homologous recombination events occurring within the long homology arm of the fneoΔ1Δ2 targeting vector either 3’ or 5’, respectively, of the deleted nPE2 sequences. The latter crossover location restored an intact nPE2 site from the wild-type chromosomal DNA. Clones with normal karyotypes (40, XY) were microinjected into e3.5 blastocysts derived from C57BL/6J mice to obtain germline-competent male chimeras. The fneoΔ1 and fneoΔ2 chimeras were both derived from J1 ES cells, while the fneoΔ1Δ2 chimeras were derived from Taffy ES cells. Chimeric males were bred to C57BL/6J females to obtain heterozygous mice. Integrity of the wild-type and mutant alleles were reconfirmed by Southern blots of tail genomic DNA and thereafter all mice were genotyped by a panel of PCR reactions specific for each mutant allele. Mice were backcrossed to the C57BL/6J strain for at least 6 generations. To obtain mice lacking the *neo* cassette early in embryogenesis, fneo mice were mated with CMV-Cre transgenic mice (B6.C-Tg(CMV-cre)1Cgn/J)[56]; The Jackson Laboratory), which express Cre recombinase in all tissues, including germ cells. Recombination in the offspring was ascertained by genomic PCR with primers flanking the floxed neo cassette, which permits discrimination of the wild-type allele and the targeted alleles lacking *neo*, while the alleles with intact *neo* cassettes are not amplified. Mice lacking both the *neo* cassette and the CMV-Cre transgene were backcrossed onto C57BL/6J for at least 6 generations.

### *In situ* Hybridization

Adult brains were rapidly extracted and fresh frozen in isopentane. Embryos were extracted, fixed overnight at 4°C in 4% PFA, and then cryoprotected in 10% sucrose in PBS overnight at 4°C. Embryos were then embedded in 10% gelatin/10% sucrose in PBS and frozen in isopentane. Tissue was cut on a cryostat (20 μm sections) and mounted on gelatin-coated slides. A 667 bp fragment encoding a portion of *Pomc* exon 3 was cloned into pGEM7. The vector was linearized with *NcoI* and served as a template for in vitro transcription and digoxigenin (DIG) labeling with T7 RNA polymerase (DIG RNA labeling kit, Roche). The resulting DIG-labeled transcript was complementary to *Pomc* mRNA. Slides with attached sections were fixed in 4% PFA in DEPC-treated PBS (DEPC-PBS) for 10 min and washed in DEPC-PBS. Sections were acetylated for 10 min with 0.01% triethanolamine, 0.02 N HCl, and 0.003% acetic anhydride and then permeabilized in 0.01% (v/v) Triton-X 100 in DEPC-PBS. After DEPC-PBS washes, sections were prehybridized in hybridization solution (50% formamide, 10% dextran sulfate, 1 mg/ml tRNA, 1X Denhardt’s solution, 30 mM NaCl, 9 mM Tris-HCl, 1 mM Tris, 5 mM NaH_2_PO_4_, 5 mM Na_2_HPO_4_, 5 mM EDTA) at room temperature for 4 hr. DIG-labeled probe was diluted in hybridization solution (750 ng/ml), heated at 80°C for 5 min, and cooled on ice. Probe was applied to slides and allowed to hybridize overnight at 72°C under coverslips. Coverslips were removed and slides were soaked in 0.2X SSC at 72°C for 45 min, then washed in 0.2X SSC at room temperature for 5 min. For detection, slides were first washed in B1 solution (100 mM Tris-HCl pH 7.5, 150 mM NaCl, 0.001% Triton-X 100) and blocked in B1 with 10% heat-inactivated goat serum (HINGS) and 100 mM lysine. Next, slides were incubated overnight with anti-DIG antibody conjugated to alkaline phosphatase (1:3,500; Roche) with 1% HINGS. Slides were washed in B1 and B2 (100 mM Tris-HCl pH 9, 100 mM NaCl, 50 mM MgCl_2_, 0.1% Tween 20). Signal was developed in B2 with NBT and BCIP substrates. The reaction was stopped with B1 and slides were coverslipped with Mowiol (Sigma). Sections were imaged on an upright Nikon 90i microscope equipped with a 10x objective (Nikon) in bright field mode with a consistent exposure time of 20 ms. Analysis was performed with NIS Elements software (Nikon). For e13.5 staining, hypothalamus and pituitary were outlined and integrated density was calculated by the software. The average integrated density of multiple sections per biological replicate (minimum 4 sections per replicate) was used to calculate means per genotype. For adult staining, images were thresholded for minimum object size and intensity, and automated counts were performed by the software.

### qRT-PCR

RNA was extracted with RNeasy columns (Qiagen) and reverse transcribed with random primers using GoScript Reverse Transcription system (Promega). A FAM-labeled *Pomc* probe (Mm00435874_m1; Life Technologies) and a VIC-labeled 18S rRNA probe (Mm03928990_g1; Life Technologies) were used in multiplex and quantitation was performed by the 2^−ΔΔCT^ method.

### Immunohistochemistry

Mice were transcardially perfused with 10% sucrose followed by 4% PFA. Brains were extracted, postfixed in 4% PFA overnight at 4°C, then cryoprotected in 30% sucrose in PBS overnight at 4°C. Brains were sectioned coronally on a freezing microtome at 30 μm thickness. In free-floating sections, endogenous peroxidase was blocked with 1% H_2_O_2_ in PBS for 30 min. Following PBS washes, sections were blocked with 1% normal goat serum and 0.1% Triton-X 100 for 1 hour. Sections were then incubated with primary antibodies at room temperature overnight (rabbit anti-ACTH, 1:5,000, National Hormone and Peptide Program; sheep anti-α-MSH, 1:25,000, gift of Dr. Jeffrey Tatro, Tufts New England Medical Center). Following PBS washes, sections were incubated with biotinylated secondary antibodies (1:1,000; Jackson ImmunoResearch), and then washed again in PBS. For detection, Vectastain ABC kit (Vector Labs) was used, followed by development with diaminobenzidine (0.5 mg/ml) in the presence of 0.01% H_2_O_2_. Slides were dehydrated and coverslipped with DPX.

### Peptide Radioimmunoassay (RIA)

Hypothalami were extracted in 0.1N HCl and assayed for POMC-derived peptides as previously described[57]. α-MSH RIA was performed with an antiserum that cross-reacts fully with des-acetyl-α-MSH, but has no cross-reactivity with ACTH, corticotropin-like intermediate peptide, or the free acid form of α-MSH that has not been amidated[57]. β-endorphin RIA was performed with an antiserum directed at β-endorphin18-25 (cross-reacts fully with β-endorphin1-31, β-endorphin1-27 and β-endorphin1-26 and 30% on a molar basis with β-lipotropic hormone; it has no cross-reactivity with ACTH or α-MSH)[57].

## Supporting Information

S1 Fig nPE1 multiple sequence alignment. Multiple sequence alignment (ClustalW) of nPE1*core* sequences from a representative variety of eutherian (placental) mammalian species. The DNA elements with similarity to NKX-binding sites (1A) and general homeodomain binding sites (1B, 1C) are shown within red squares. Nucleotide residues identical to the human sequence are highlighted in blue or yellow.
(TIF)

S2 Fig nPE2 multiple sequence alignment. Multiple sequence alignment (ClustalW) of regions 1 and 3 of nPE2 (as defined in ref. [29]) from a representative variety of mammalian species. The species include placental mammals as well as one marsupial (opossum, *Monodelphis domestica*) and a monotreme (platypus, *Ornithorhynchus anatinus*). The DNA elements with similarity to NKX-binding sites (2.1C, 2.3C) and general homeodomain binding sites (2.1A-B, 2.3A-B) are shown within red squares. Nucleotide residues identical in a majority of species are highlighted in blue or yellow.
(TIF)

S3 Fig Mutated *Pomc* alleles. (A) Schematic diagrams of the mutated *Pomc* alleles after homologous recombination in ES cells and prior to germline excision of the LoxP-flanked (orange arrowheads) *neo* cassettes by crosses of heterozygous fneo mice to CMV-Cre mice. *EcoRV* restriction sites and resulting fragment sizes are indicated. Southern blot probes are shown bound at their targets (see probe legend). Thick gray lines indicate portions of the targeting constructs with homology to the wild-type (+) allele, while thick black lines indicate endogenous genomic DNA sequences. (B) Southern blot verification of appropriate DNA fragment sizes following *EcoRV* digestion of genomic DNA extracted from mice of each genotype.
(TIF)

S4 Fig POMC-derived peptides in nPE mutant mice. (A, C) α-MSH and (B, D) β-endorphin content in brain areas innervated by POMC neuron terminals. Amygdala (amyg), thalamus (thal), and brainstem (bstem). n = 6–8 of each genotype. (E) α-MSH content in anterior pituitary. n = 6–12 of each genotype. (F) Representative α-MSH immunohistochemistry in coronal hypothalamic sections. The specific labeling for α-MSH is predominantly in POMC fibers rather than POMC neuronal soma as shown for ACTH immunohistochemistry in Fig. 4A. Scale bar, 200 μm. Quantitative data were obtained by radioimmunoassay and are presented as mean + 1 S.E.M. Genotype means were compared by two-tailed t-tests. ** *P* < 0.01, *** *P* < 0.001 compared to +/+.
(TIF)

S5 Fig Reproductive phenotype of nPE mutant mice. (A-C) Homozygous male +/+, Δ1/Δ1, or Δ2/Δ2 mice were housed in trios with female mice of the same genotype (age 8 wk; n = 4–6 trios per genotype). Latency to parturition illustrated by a Kaplan-Meier plot (A), litter size (B), and newborn pup weight (C) were recorded for the first litter from each dam. Data are presented as mean + 1 S.E.M. Genotype means were compared by two-tailed t-tests. There were no significant differences between genotypes.
(TIF)

S6 Fig Supplementary metabolic and behavioral phenotypes of nPE mutant mice. See genotype color key at the top of the figure. (A) Intraperitoneal glucose tolerance test. n = 4–6. (B) Morning plasma corticosterone in unstressed (BASAL) or 20 min restraint-stressed (STRESS) mice. n = 3–9. (C) Food intake of mice presented with a free choice of unlimited low fat (LFD; 10% kcal fat) or high fat (HFD; 60% kcal fat) diets for 24 hr. n = 3–5. (D) 1 hr food intake at the onset of the dark cycle in the absence of stress (CTRL) or, on a different test day, immediately following 20 min restraint stress. n = 3–5. (E) Hypothalamic *Pomc* expression in mice fed ad libitum or fasted overnight (16 h). n = 5–12 of each genotype/treatment combination. (F) Feeding latency in a novel open field environment after a 16 hr fast. n = 3. (G-J) Mice were placed in CLAMS automated metabolic chambers for 72 hr, and oxygen consumption (VO2; G), respiratory exchange ratio (RER; H), horizontal activity (I) and rearing activity (J) were measured. n = 8. (K) Mice were given ad libitum access to food for 4 hr daily during the light cycle and daily food intake was recorded. n = 3–4. (L) Systolic blood pressure (BP) and (M) heart rate were measured by tail cuff. n = 4–6. (N, O) Mice fed ad libitum (N) or fasted overnight (16 hr; O) were housed at 4°C and rectal temperature was measured hourly. n = 4. Data are presented as mean ± 1 S.E.M. Group means were compared by two-tailed t-tests. ** P < 0.01, *** P < 0.001.
(TIF)

## References

[1] Levine M (2010) Transcriptional enhancers in animal development and evolution. Curr Biol 20: R754–63. 20833320 10.1016/j.cub.2010.06.070 PMC4280268

[2] Rubinstein M, de Souza FSJ (2013) Evolution of transcriptional enhancers and animal diversity. Philos Trans R Soc Lond B Biol Sci 368: 20130017 24218630 10.1098/rstb.2013.0017 PMC3826491

[3] Shen Y, Yue F, McCleary DF, Ye Z, Edsall L, et al. (2012) A map of the cis-regulatory sequences in the mouse genome. Nature 488: 116–120. 22763441 10.1038/nature11243 PMC4041622

[4] Thurman RE, Rynes E, Humbert R, Vierstra J, Maurano MT, et al. (2012) The accessible chromatin landscape of the human genome. Nature 489: 75–82. 22955617 10.1038/nature11232 PMC3721348

[5] Sanyal A, Lajoie BR, Jain G, Dekker J (2012) The long-range interaction landscape of gene promoters. Nature 489: 109–113. 22955621 10.1038/nature11279 PMC3555147

[6] Andersson R, Gebhard C, Miguel-Escalada I, Hoof I, Bornholdt J, et al. (2014) An atlas of active enhancers across human cell types and tissues. Nature 507: 455–461. 10.1038/nature12787 24670763 PMC5215096

[7] Goto T, Macdonald P, Maniatis T (1989) Early and late periodic patterns of even skipped expression are controlled by distinct regulatory elements that respond to different spatial cues. Cell 57: 413–422. 2720776 10.1016/0092-8674(89)90916-1

[8] Yuh CH, Davidson EH (1996) Modular cis-regulatory organization of Endo16, a gut-specific gene of the sea urchin embryo. Development 122: 1069–1082. 8620834 10.1242/dev.122.4.1069

[9] Jeong Y, El-Jaick K, Roessler E, Muenke M, Epstein DJ (2006) A functional screen for sonic hedgehog regulatory elements across a 1 Mb interval identifies long-range ventral forebrain enhancers. Development 133: 761–772. 16407397 10.1242/dev.02239

[10] Werner T, Hammer A, Wahlbuhl M, Bösl MR, Wegner M (2007) Multiple conserved regulatory elements with overlapping functions determine Sox10 expression in mouse embryogenesis. Nucleic Acids Res 35: 6526–6538. 17897962 10.1093/nar/gkm727 PMC2095789

[11] Wilkins AS (1997) Canalization: a molecular genetic perspective. Bioessays 19: 257–262. 9080776 10.1002/bies.950190312

[12] Diss G, Ascencio D, Deluna A, Landry CR (2013) Molecular mechanisms of paralogous compensation and the robustness of cellular networks. J Exp Zool B Mol Dev Evol. 10.1002/jez.b.22555 24376223

[13] Nowak MA, Boerlijst MC, Cooke J, Smith JM (1997) Evolution of genetic redundancy. Nature 388: 167–171. 9217155 10.1038/40618

[14] Barbaric I, Miller G, Dear TN (2007) Appearances can be deceiving: phenotypes of knockout mice. Brief Funct Genomic Proteomic 6: 91–103. 17584761 10.1093/bfgp/elm008

[15] Brookfield JF (1997) Genetic redundancy. Adv Genet 36: 137–155. 9348654 10.1016/s0065-2660(08)60308-9

[16] Zhang J (2012) Genetic redundancies and their evolutionary maintenance. Adv Exp Med Biol 751: 279–300. 22821463 10.1007/978-1-4614-3567-9_13

[17] Hong J-W, Hendrix DA, Levine MS (2008) Shadow enhancers as a source of evolutionary novelty. Science 321: 1314 10.1126/science.1160631 18772429 PMC4257485

[18] Hobert O (2010) Gene regulation: enhancers stepping out of the shadow. Curr Biol 20: R697–9. 10.1016/j.cub.2010.07.035 20833307

[19] Barolo S (2012) Shadow enhancers: frequently asked questions about distributed cis-regulatory information and enhancer redundancy. Bioessays 34: 135–141. 10.1002/bies.201100121 22083793 PMC3517143

[20] Frankel N, Davis GK, Vargas D, Wang S, Payre F, et al. (2010) Phenotypic robustness conferred by apparently redundant transcriptional enhancers. Nature 466: 490–493. 10.1038/nature09158 20512118 PMC2909378

[21] Perry MW, Boettiger AN, Bothma JP, Levine M (2010) Shadow enhancers foster robustness of Drosophila gastrulation. Curr Biol 20: 1562–1567. 10.1016/j.cub.2010.07.043 20797865 PMC4257487

[22] Perry MW, Boettiger AN, Levine M (2011) Multiple enhancers ensure precision of gap gene-expression patterns in the Drosophila embryo. Proc Natl Acad Sci U S A 108: 13570–13575. 10.1073/pnas.1109873108 21825127 PMC3158186

[23] Waddington CH (1942) Canalization of development and the inheritance of acquired characters. Nature 150: 563–565. 13666847 10.1038/1831654a0

[24] Yaswen L, Diehl N, Brennan MB, Hochgeschwender U (1999) Obesity in the mouse model of pro-opiomelanocortin deficiency responds to peripheral melanocortin. Nat Med 5: 1066–1070. 10470087 10.1038/12506

[25] Krude H, Biebermann H, Luck W, Horn R, Brabant G, et al. (1998) Severe early-onset obesity, adrenal insufficiency and red hair pigmentation caused by POMC mutations in humans. Nat Genet 19: 155–157. 9620771 10.1038/509

[26] Bumaschny VF, Yamashita M, Casas-Cordero R, Otero-Corchón V, de Souza FSJ, et al. (2012) Obesity-programmed mice are rescued by early genetic intervention. J Clin Invest 122: 4203–4212. 10.1172/JCI62543 23093774 PMC3484438

[27] De Souza FSJ, Santangelo AM, Bumaschny V, Avale ME, Smart JL, et al. (2005) Identification of neuronal enhancers of the proopiomelanocortin gene by transgenic mouse analysis and phylogenetic footprinting. Mol Cell Biol 25: 3076–3086. 15798195 10.1128/MCB.25.8.3076-3086.2005 PMC1069613

[28] Franchini LF, López-Leal R, Nasif S, Beati P, Gelman DM, et al. (2011) Convergent evolution of two mammalian neuronal enhancers by sequential exaptation of unrelated retroposons. Proc Natl Acad Sci U S A 108: 15270–15275. 10.1073/pnas.1104997108 21876128 PMC3174587

[29] Santangelo AM, de Souza FSJ, Franchini LF, Bumaschny VF, Low MJ, et al. (2007) Ancient exaptation of a CORE-SINE retroposon into a highly conserved mammalian neuronal enhancer of the proopiomelanocortin gene. PLoS Genet 3: 1813–1826. 17922573 10.1371/journal.pgen.0030166 PMC2000970

[30] Langlais D, Couture C, Sylvain-Drolet G, Drouin J (2011) A pituitary-specific enhancer of the POMC gene with preferential activity in corticotrope cells. Mol Endocrinol 25: 348–359. 10.1210/me.2010-0422 21193556 PMC5417307

[31] Elkabes S, Loh YP, Nieburgs A, Wray S (1989) Prenatal ontogenesis of pro-opiomelanocortin in the mouse central nervous system and pituitary gland: an in situ hybridization and immunocytochemical study. Brain Res Dev Brain Res 46: 85–95. 2706773 10.1016/0165-3806(89)90145-4

[32] Japón MA, Rubinstein M, Low MJ (1994) In situ hybridization analysis of anterior pituitary hormone gene expression during fetal mouse development. J Histochem Cytochem 42: 1117–1125. 8027530 10.1177/42.8.8027530

[33] Zhan C, Zhou J, Feng Q, Zhang J-E, Lin S, et al. (2013) Acute and long-term suppression of feeding behavior by POMC neurons in the brainstem and hypothalamus, respectively. J Neurosci 33: 3624–3632. 10.1523/JNEUROSCI.2742-12.2013 23426689 PMC6619547

[34] Conant GC, Wolfe KH (2008) Turning a hobby into a job: how duplicated genes find new functions. Nat Rev Genet 9: 938–950. 10.1038/nrg2482 19015656

[35] De Souza FSJ, Nasif S, López-Leal R, Levi DH, Low MJ, et al. (2011) The estrogen receptor α colocalizes with proopiomelanocortin in hypothalamic neurons and binds to a conserved motif present in the neuron-specific enhancer nPE2. Eur J Pharmacol 660: 181–187. 10.1016/j.ejphar.2010.10.114 21211522 PMC3097136

[36] Smart JL, Tolle V, Low MJ (2006) Glucocorticoids exacerbate obesity and insulin resistance in neuron-specific proopiomelanocortin-deficient mice. J Clin Invest 116: 495–505. 16440060 10.1172/JCI25243 PMC1350998

[37] Padilla SL, Carmody JS, Zeltser LM (2010) Pomc-expressing progenitors give rise to antagonistic neuronal populations in hypothalamic feeding circuits. Nat Med 16: 403–405. 10.1038/nm.2126 20348924 PMC2854504

[38] Dvornyk V, Vinogradova O, Nevo E (2003) Origin and evolution of circadian clock genes in prokaryotes. Proc Natl Acad Sci U S A 100: 2495–2500. 12604787 10.1073/pnas.0130099100 PMC151369

[39] Speakman JR (2013) Evolutionary perspectives on the obesity epidemic: adaptive, maladaptive, and neutral viewpoints. Annu Rev Nutr 33: 289–317. 10.1146/annurev-nutr-071811-150711 23862645

[40] Hammoud AO, Gibson M, Peterson CM, Meikle AW, Carrell DT (2008) Impact of male obesity on infertility: a critical review of the current literature. Fertil Steril 90: 897–904. 10.1016/j.fertnstert.2008.08.026 18929048

[41] Brewer CJ, Balen AH (2010) The adverse effects of obesity on conception and implantation. Reproduction 140: 347–364. 20395425 10.1530/REP-09-0568

[42] Sakabe NJ, Nobrega MA (2013) Beyond the ENCODE project: using genomics and epigenomics strategies to study enhancer evolution. Philos Trans R Soc Lond B Biol Sci 368: 20130022 10.1098/rstb.2013.0022 24218635 PMC3826496

[43] Sakabe NJ, Savic D, Nobrega MA (2012) Transcriptional enhancers in development and disease. Genome Biol 13: 238. 22269347 10.1186/gb-2012-13-1-238 PMC3334578

[44] Smemo S, Tena JJ, Kim K-H, Gamazon ER, Sakabe NJ, et al. (2014) Obesity-associated variants within FTO form long-range functional connections with IRX3. Nature 507: 371–375. 10.1038/nature13138 24646999 PMC4113484

[45] Graff M, Ngwa JS, Workalemahu T, Homuth G, Schipf S, et al. (2013) Genome-wide analysis of BMI in adolescents and young adults reveals additional insight into the effects of genetic loci over the life course. Hum Mol Genet 22: 3597–3607. 10.1093/hmg/ddt205 23669352 PMC3736869

[46] Cousminer DL, Berry DJ, Timpson NJ, Ang W, Thiering E, et al. (2013) Genome-wide association and longitudinal analyses reveal genetic loci linking pubertal height growth, pubertal timing and childhood adiposity. Hum Mol Genet 22: 2735–2747. 10.1093/hmg/ddt104 23449627 PMC3674797

[47] Speliotes EK, Willer CJ, Berndt SI, Monda KL, Thorleifsson G, et al. (2010) Association analyses of 249,796 individuals reveal 18 new loci associated with body mass index. Nat Genet 42: 937–948. 20935630 10.1038/ng.686 PMC3014648

[48] Queitsch C, Carlson KD, Girirajan S (2012) Lessons from model organisms: phenotypic robustness and missing heritability in complex disease. PLoS Genet 8: e1003041 10.1371/journal.pgen.1003041 23166511 PMC3499356

[49] Symmons O, Uslu VV, Tsujimura T, Ruf S, Nassari S, et al. (2014) Functional and topological characteristics of mammalian regulatory domains. Genome Res 24: 390–400. 10.1101/gr.163519.113 24398455 PMC3941104

[50] Marinić M, Aktas T, Ruf S, Spitz F (2013) An integrated holo-enhancer unit defines tissue and gene specificity of the Fgf8 regulatory landscape. Dev Cell 24: 530–542. 10.1016/j.devcel.2013.01.025 23453598

[51] Mali P, Yang L, Esvelt KM, Aach J, Guell M, et al. (2013) RNA-guided human genome engineering via Cas9. Science 339: 823–826. 23287722 10.1126/science.1232033 PMC3712628

[52] Gaj T, Gersbach CA, Barbas CF (2013) ZFN, TALEN, and CRISPR/Cas-based methods for genome engineering. Trends Biotechnol 31: 397–405. 23664777 10.1016/j.tibtech.2013.04.004 PMC3694601

[53] Larkin MA, Blackshields G, Brown NP, Chenna R, McGettigan PA, et al. (2007) Clustal W and Clustal X version 2.0. Bioinformatics 23: 2947–2948. 17846036 10.1093/bioinformatics/btm404

[54] Cowley MA, Smart JL, Rubinstein M, Cerdán MG, Diano S, et al. (2001) Leptin activates anorexigenic POMC neurons through a neural network in the arcuate nucleus. Nature 411: 480–484. 11373681 10.1038/35078085

[55] Li E, Bestor TH, Jaenisch R (1992) Targeted mutation of the DNA methyltransferase gene results in embryonic lethality. Cell 69: 915–926. 1606615 10.1016/0092-8674(92)90611-f

[56] Schwenk F, Baron U, Rajewsky K (1995) A cre-transgenic mouse strain for the ubiquitous deletion of loxP-flanked gene segments including deletion in germ cells. Nucleic Acids Res 23: 5080–5081. 8559668 10.1093/nar/23.24.5080 PMC307516

[57] Savontaus E, Breen TL, Kim A, Yang LM, Chua SC, et al. (2004) Metabolic effects of transgenic melanocyte-stimulating hormone overexpression in lean and obese mice. Endocrinology 145: 3881–3891. 15117873 10.1210/en.2004-0263

