# Correction: Partially Redundant Enhancers Cooperatively Maintain Mammalian *Pomc* Expression Above a Critical Functional Threshold

**DOI:** 10.1371/journal.pgen.1005133

**Published:** 2015-04-10

**Authors:** The PLOS Genetics Staff

The corresponding author information is incomplete. Marcelo Rubinstein (mrubins@dna.uba.ar) and Malcolm J. Low (mjlow@umich.edu) are co-corresponding authors for this article.
