# Supplementary figures and images for: Partially Redundant Enhancers Cooperatively Maintain Mammalian Pomc Expression Above a Critical Functional Threshold
Source: PLoS Genet. 2015 Feb 11;11(2):e1004935. doi: 10.1371/journal.pgen.1004935 (PMC4335486; doi:10.1371/journal.pgen.1004935)

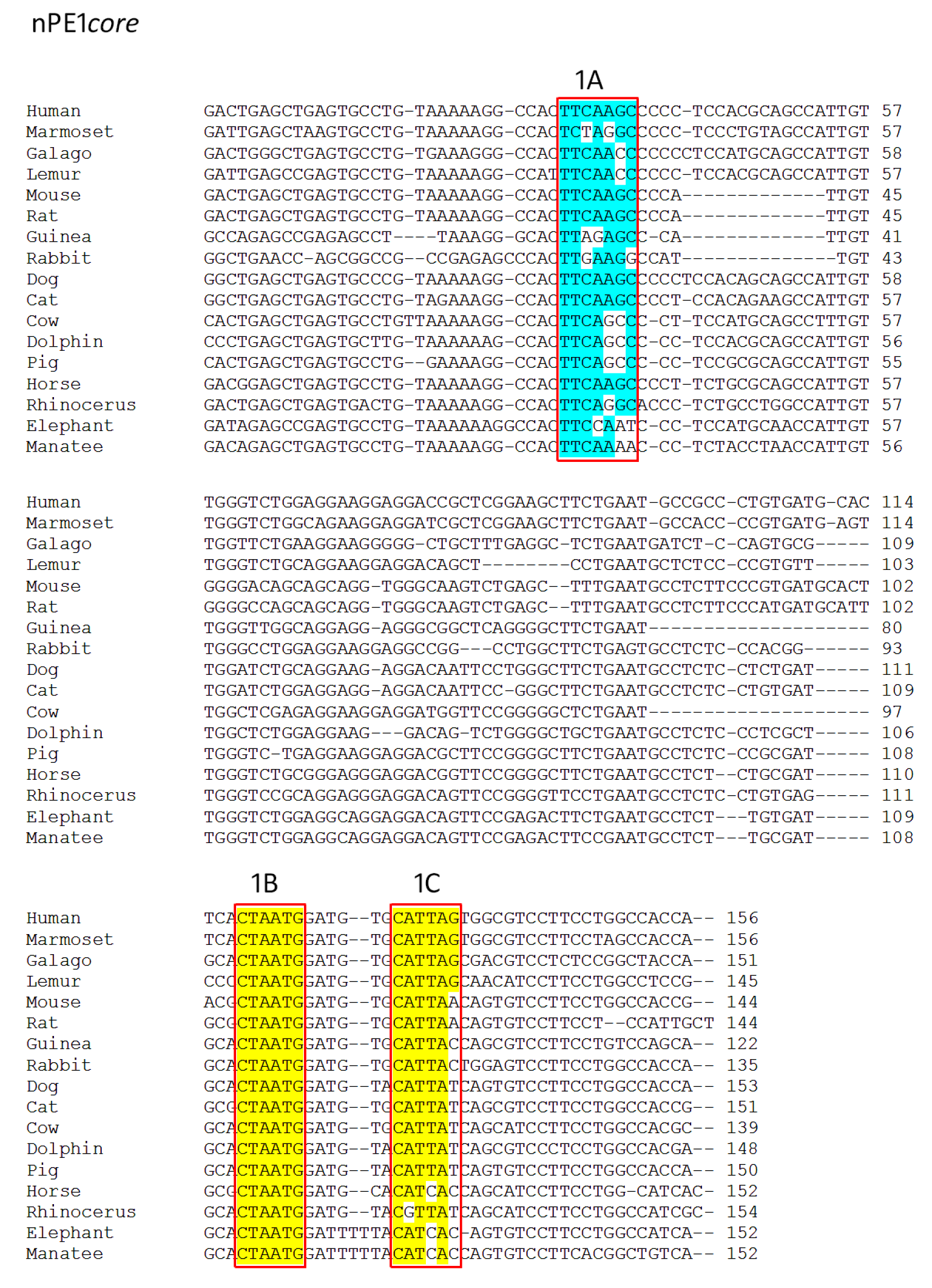

Supplement: S1 Fig — Multiple sequence alignment (ClustalW) of nPE1core sequences from a representative variety of eutherian (placental) mammalian species. The DNA elements with similarity to NKX-binding sites (1A) and general homeodomain binding sites (1B, 1C) are shown within red squares. Nucleotide residues identical to the human sequence are highlighted in blue or yellow. (TIF) [file pgen.1004935.s001.tif]

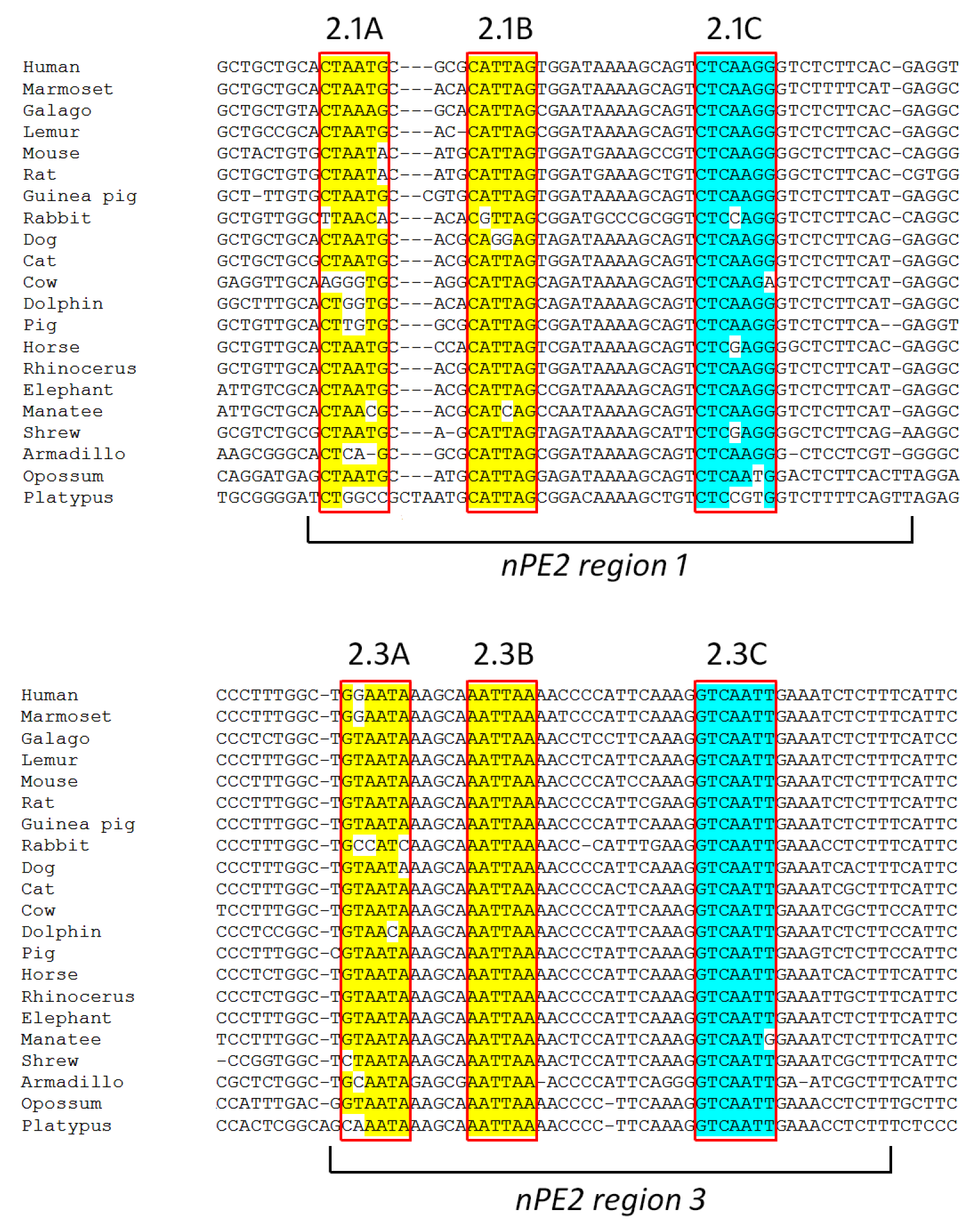

Supplement: S2 Fig — Multiple sequence alignment (ClustalW) of regions 1 and 3 of nPE2 (as defined in ref. [29]) from a representative variety of mammalian species. The species include placental mammals as well as one marsupial (opossum, Monodelphis domestica) and a monotreme (platypus, Ornithorhynchus anatinus). The DNA elements with similarity to NKX-binding sites (2.1C, 2.3C) and general homeodomain binding sites (2.1A-B, 2.3A-B) are shown within red squares. Nucleotide residues identical in a majority of species are highlighted in blue or yellow. (TIF) [file pgen.1004935.s002.tif]

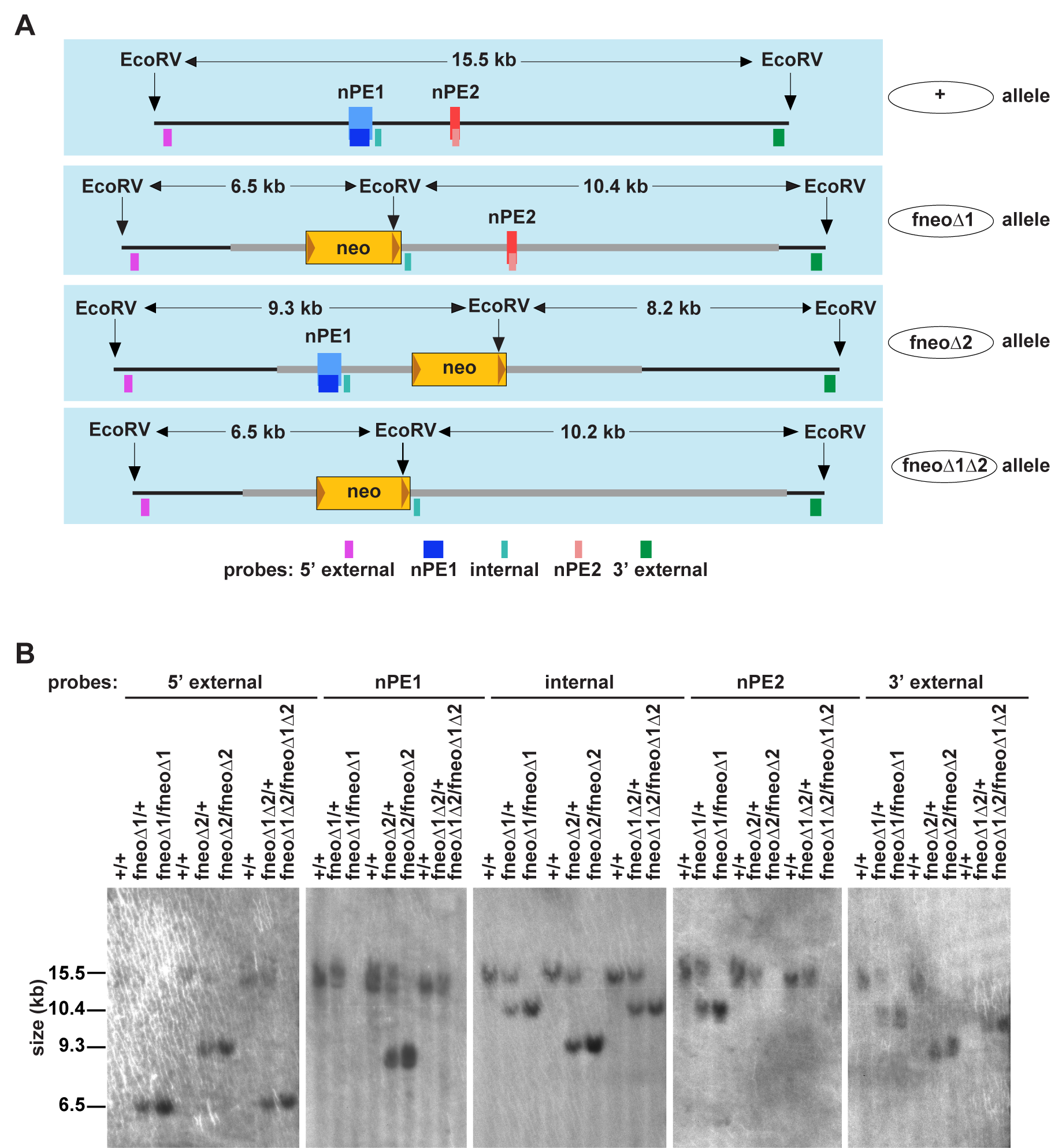

Supplement: S3 Fig — (A) Schematic diagrams of the mutated Pomc alleles after homologous recombination in ES cells and prior to germline excision of the LoxP-flanked (orange arrowheads) neo cassettes by crosses of heterozygous fneo mice to CMV-Cre mice. EcoRV restriction sites and resulting fragment sizes are indicated. Southern blot probes are shown bound at their targets (see probe legend). Thick gray lines indicate portions of the targeting constructs with homology to the wild-type (+) allele, while thick black lines indicate endogenous genomic DNA sequences. (B) Southern blot verification of appropriate DNA fragment sizes following EcoRV digestion of genomic DNA extracted from mice of each genotype. (TIF) [file pgen.1004935.s003.tif]

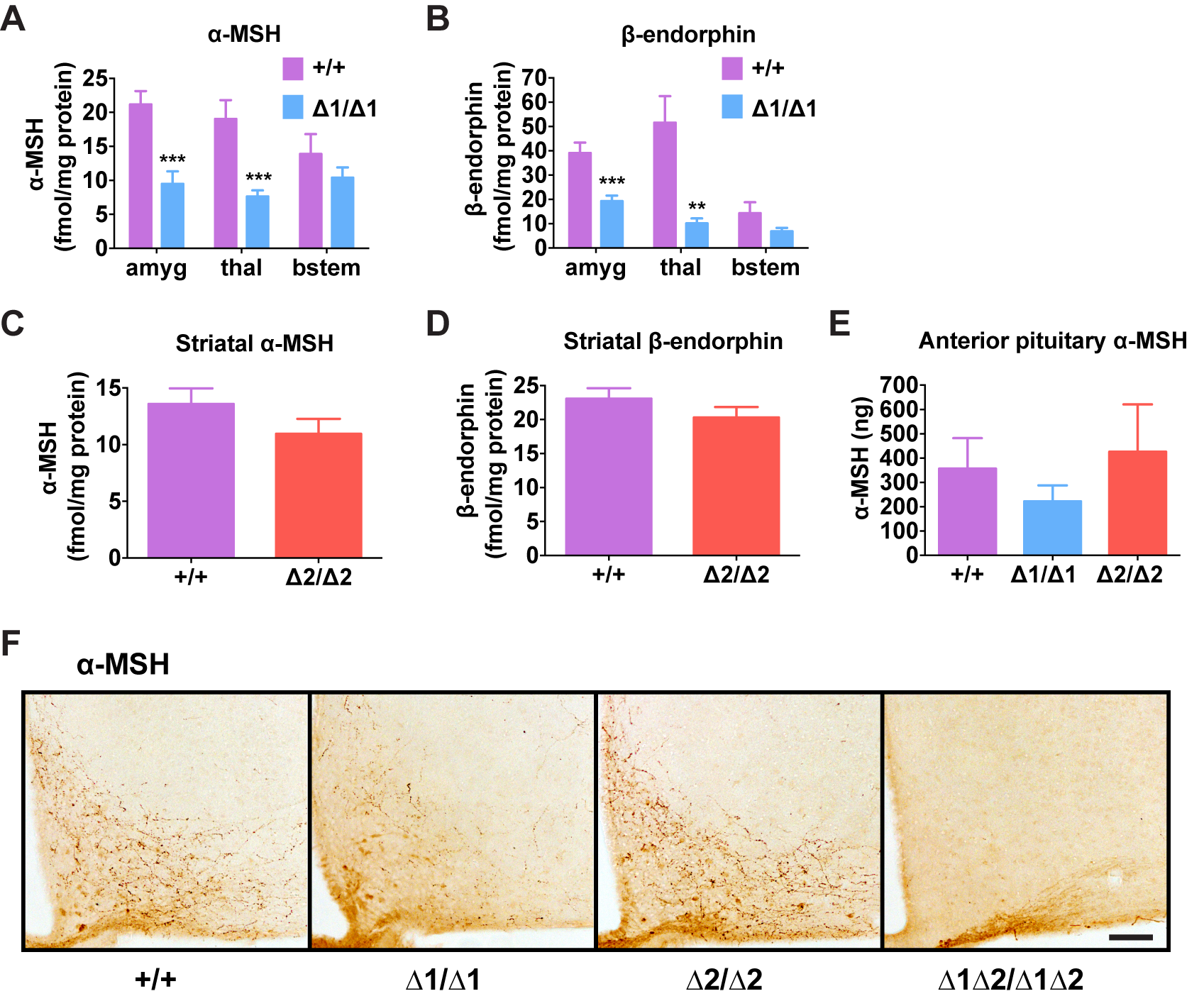

Supplement: S4 Fig — (A, C) α-MSH and (B, D) β-endorphin content in brain areas innervated by POMC neuron terminals. Amygdala (amyg), thalamus (thal), and brainstem (bstem). n = 6–8 of each genotype. (E) α-MSH content in anterior pituitary. n = 6–12 of each genotype. (F) Representative α-MSH immunohistochemistry in coronal hypothalamic sections. The specific labeling for α-MSH is predominantly in POMC fibers rather than POMC neuronal soma as shown for ACTH immunohistochemistry in Fig. 4A. Scale bar, 200 μm. Quantitative data were obtained by radioimmunoassay and are presented as mean + 1 S.E.M. Genotype means were compared by two-tailed t-tests. ** P < 0.01, *** P < 0.001 compared to +/+. (TIF) [file pgen.1004935.s004.tif]

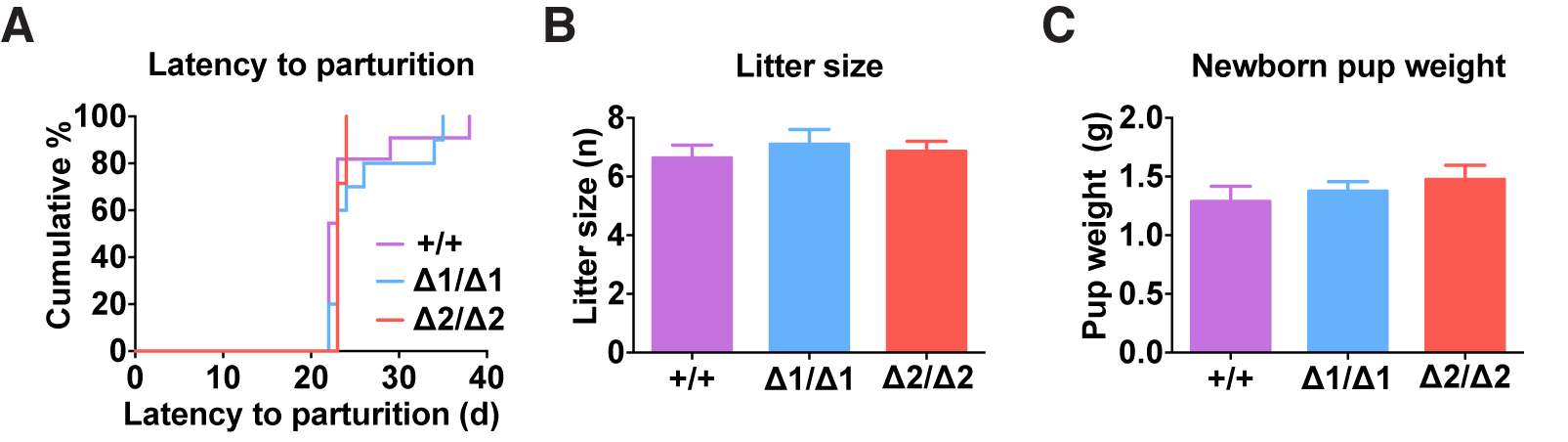

Supplement: S5 Fig — (A-C) Homozygous male +/+, Δ1/Δ1, or Δ2/Δ2 mice were housed in trios with female mice of the same genotype (age 8 wk; n = 4–6 trios per genotype). Latency to parturition illustrated by a Kaplan-Meier plot (A), litter size (B), and newborn pup weight (C) were recorded for the first litter from each dam. Data are presented as mean + 1 S.E.M. Genotype means were compared by two-tailed t-tests. There were no significant differences between genotypes. (TIF) [file pgen.1004935.s005.tif]

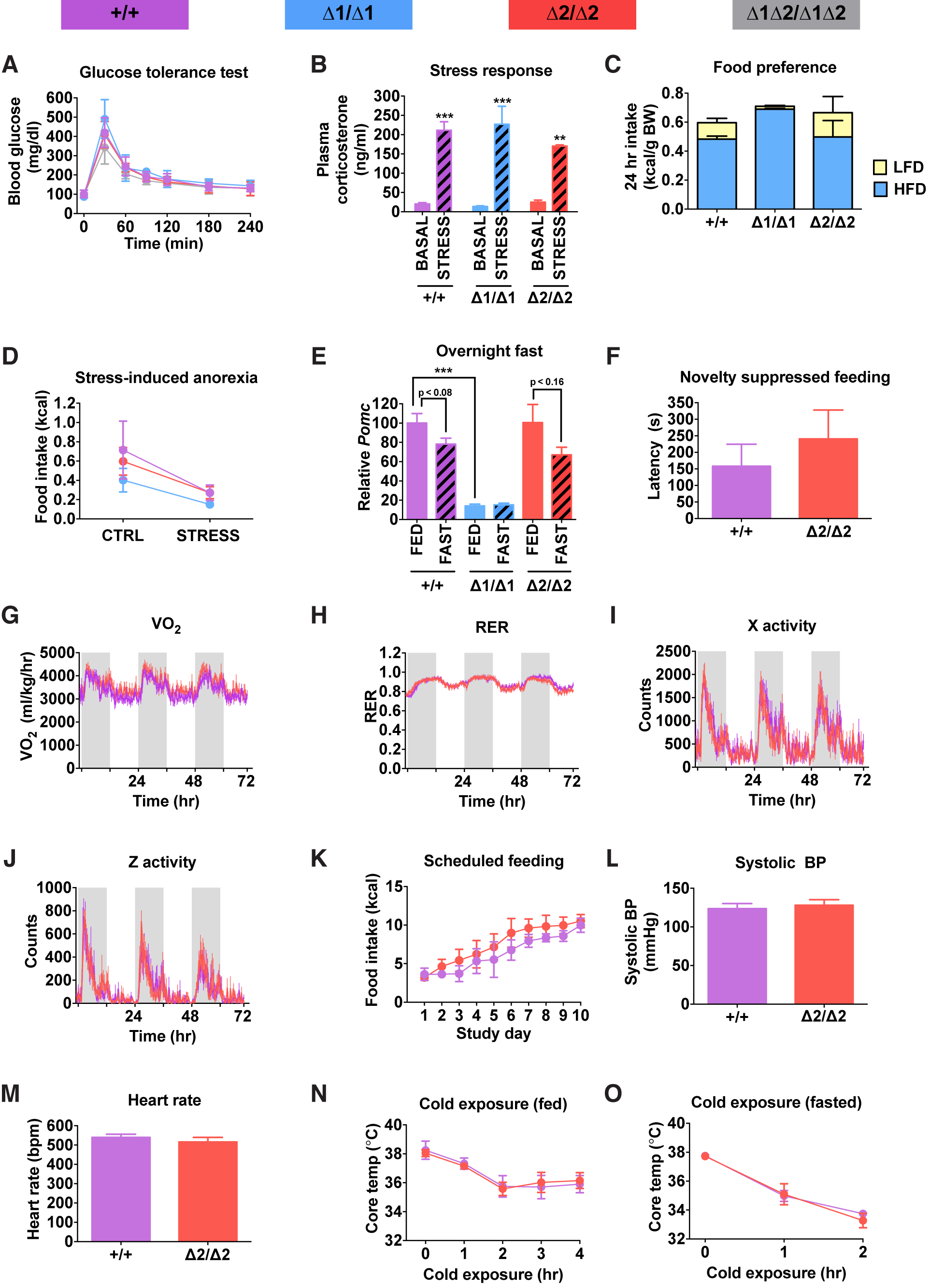

Supplement: S6 Fig — See genotype color key at the top of the figure. (A) Intraperitoneal glucose tolerance test. n = 4–6. (B) Morning plasma corticosterone in unstressed (BASAL) or 20 min restraint-stressed (STRESS) mice. n = 3–9. (C) Food intake of mice presented with a free choice of unlimited low fat (LFD; 10% kcal fat) or high fat (HFD; 60% kcal fat) diets for 24 hr. n = 3–5. (D) 1 hr food intake at the onset of the dark cycle in the absence of stress (CTRL) or, on a different test day, immediately following 20 min restraint stress. n = 3–5. (E) Hypothalamic Pomc expression in mice fed ad libitum or fasted overnight (16 h). n = 5–12 of each genotype/treatment combination. (F) Feeding latency in a novel open field environment after a 16 hr fast. n = 3. (G-J) Mice were placed in CLAMS automated metabolic chambers for 72 hr, and oxygen consumption (VO2; G), respiratory exchange ratio (RER; H), horizontal activity (I) and rearing activity (J) were measured. n = 8. (K) Mice were given ad libitum access to food for 4 hr daily during the light cycle and daily food intake was recorded. n = 3–4. (L) Systolic blood pressure (BP) and (M) heart rate were measured by tail cuff. n = 4–6. (N, O) Mice fed ad libitum (N) or fasted overnight (16 hr; O) were housed at 4°C and rectal temperature was measured hourly. n = 4. Data are presented as mean ± 1 S.E.M. Group means were compared by two-tailed t-tests. ** P < 0.01, *** P < 0.001. (TIF) [file pgen.1004935.s006.tif]
